# A Fragment-Based Approach for the Development of G-Quadruplex Ligands: Role of the Amidoxime Moiety

**DOI:** 10.3390/molecules23081874

**Published:** 2018-07-27

**Authors:** Martina Tassinari, Alberto Lena, Elena Butovskaya, Valentina Pirota, Matteo Nadai, Mauro Freccero, Filippo Doria, Sara N. Richter

**Affiliations:** 1 Department of Molecular Medicine, University of Padua, via A. Gabelli 63, 35121 Padua, Italy; martina.tassinari@unipd.it (M.T.); elena.butovskaya@gmail.com (E.B.); matteo.nadai@unipd.it (M.N.); 2 Department of Chemistry, University of Pavia, Viale Taramelli 10, 27100 Pavia, Italy; alberto.lena01@ateneopv.it (A.L.); valentina.pirota01@universitadipavia.it (V.P.); mauro.freccero@unipv.it (M.F.)

**Keywords:** G-quadruplex, amidoximes/oxadiazole/pyridine, fragments, FBA, FRET-melting

## Abstract

G-quadruplex (G4) nucleic acid structures have been reported to be involved in several human pathologies, including cancer, neurodegenerative disorders and infectious diseases; however, G4 targeting compounds still need implementation in terms of drug-like properties and selectivity in order to reach the clinical use. So far, G4 ligands have been mainly identified through high-throughput screening methods or design of molecules with pre-set features. Here, we describe the development of new heterocyclic ligands through a fragment-based drug discovery (FBDD) approach. The ligands were designed against the major G4 present in the long terminal repeat (LTR) promoter region of the human immunodeficiency virus-1 (HIV-1), the stabilization of which has been shown to suppress viral gene expression and replication. Our method is based on the generation of molecular fragment small libraries, screened against the target to further elaborate them into lead compounds. We screened 150 small molecules, composed by structurally and chemically different fragments, selected from commercially available and in-house compounds; synthetic elaboration yielded several G4 ligands and two final G4 binders, both embedding an amidoxime moiety; one of these two compounds showed preferential binding for the HIV-1 LTR G4. This work presents the discovery of a novel potential pharmacophore and highlights the possibility to apply a fragment-based approach to develop G4 ligands with unexpected chemical features.

## 1. Introduction

**Figure 1:**
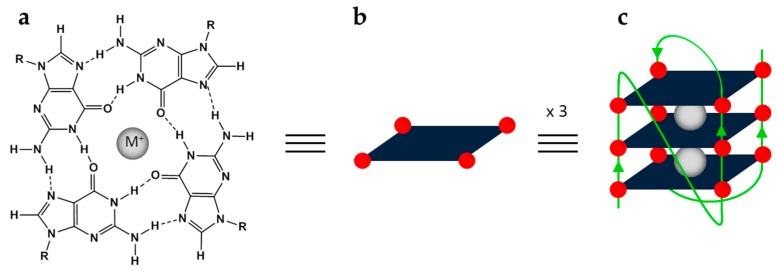
The G-quadruplex structure. (**a**) Chemical structure and (**b**) schematic illustration of a G-tetrad, composed of four guanines linked together through Hoogsteen H-bonds; M^+^ represents the monovalent cation coordinated at the centre of the tetrad. (**c**) Example of intramolecular G4 structure.

G-quadruplexes (G4s) are nucleic acids secondary structures that may form in single-stranded guanine (G)-rich sequences under physiological conditions [1,2,3]. Four Gs bind via Hoogsteen-type hydrogen bonds base-pairing to yield G-quartets, which in turn stack on top of each other to form the G4 (Figure 1). G4s are highly polymorphic, both in terms of strand stoichiometry (forming both inter- and intramolecular structures) and strand orientation/topology. The presence of K^+^ cations specifically supports G4 formation and stability [4,5,6]. In the human genome G4 DNA motifs have been found in telomeres, G-rich micro- and mini-satellites, up-stream to oncogene promoters and within the ribosomal DNA (rDNA) [7,8,9,10,11,12]. Human G4 DNA motifs are over-expressed in recombinogenic regions [13,14,15], which are associated with genomic damage in cancer cells. Additionally, these regions show mutational patterns that preserve the potential to form G4 DNA structures [11]. The identification of G4 binding proteins [16,17,18] and G4 visualization in cells with antibody-based technology [19,20] have also provided convincing evidence of the existence of cellular G4s in vivo, prevalently in tumours compared to normal tissues [21]. More recently, research on G4s has also focused on prokaryotes [22] and viruses. Indeed, G4s have been found to control key viral steps [23,24,25,26] and treatment with G4 ligands has shown to impair viral replication [27]. Due to the possibility to target pathogenic pathways by stabilizing G4 structures, several G4 binders have been developed. In particular, ligands targeting tumour mechanisms at the telomere and oncogene promoter level have been reported [24]. These share an aromatic core and protonable side chains. Some of these compounds showed interesting antitumor properties; nevertheless, only quarfloxin proceeded into Phase II clinical trials. Unfortunately, its limited bioavailability prevented further progress [24]. The success of tumour targeting through a G4-binding mechanism heavily relies on the sustainable identification of new and selective chemical entities. 

Fragment-based drug discovery (FBDD) is a validated drug design strategy and a successful alternative to traditional high-throughput screening methods [28]. Fragments are defined by “the rule of three” (Ro3) [29] as organic molecules with molecular weight (MW) in the range of 100–300 Da [30], each with a limited number (<3) of hydrogen bond donors and acceptors and rotatable bonds. Following detection, fragment hits can be expanded or combined to generate larger molecules with high affinity, selectivity and more drug-like properties [31]. As the concept of fragment-based lead generation has become established, it has also emerged that integrated approaches with fragment-based hits, such as high-throughput screening (HTS) can be very fruitful [32]. Using this approach, the drug vemurafenib was the first clinical candidate born out of a fragment-screening program approved by the US Food and Drug Administration in 2011 for the treatment of metastatic melanoma [33]. Several other fragment-derived molecules are now at the clinical stage [30].

A fragment-based approach has recently been applied to target G4 in the c-MYC promoter and inhibition of c-MYC expression has been obtained, even though at fragment concentrations in the μM range [34]. Selectivity towards RNA vs. DNA G4s has also been achieved by fragment expansion by click chemistry [35].

Here the FBDD approach was used to develop new G4 ligands targeting the major DNA G4 structure present in the Human immunodeficiency virus type-1 (HIV-1) LTR (Long Terminal Repeat). We have previously demonstrated that three mutually exclusive and functionally significant G4s can fold within the HIV-1 LTR, that is, LTR-II, LTR-III and LTR-IV, with LTR-III being the predominant structure [26]. LTR G4s act as negative regulators of viral transcription and their stabilization by G4 ligands leads to a remarkable antiviral effect, thus validating the role of G4s as anti-HIV-1 target [25,36]. In this context, the final goal of the present work was to identify selective and efficient ligands for LTR-III, characterized by a new pharmacophore unit with favourable drug-like properties. We preliminarily generated small libraries of molecular fragments, screening them against the target. The resulting hits were synthetically further elaborated into lead compounds, progressively implementing the structural complexity of the aryl- and heteroaryl-scaffold, keeping unmodified the structural moiety identified at the preliminary pre-screening level.

## 2. Results and Discussion

### 2.1. Initial Screening of Low-Molecular Weight Fragment Molecules

We initially screened around 150 structurally and chemically different fragment molecules, selected from an in-house library of commercially available or in-lab synthesized compounds. Each member of this starting fragment family obeyed the principal criterion of fragment libraries, that is, Ro3. All the fragments were ≥95% pure and showed >1 mM aqueous solubility. Their interaction with two G4 forming sequences was investigated: in particular, with the HIV-1 LTR-III sequence [26], which modulates HIV-1 promoter activity and with the human telomeric (hTel) [37], which is likely the most common and frequent G4 structure within human genome. Additionally, the two model G4s, folding into different G4 conformations, allow to detect conformation-specific hits. A double stranded (ds) oligonucleotide was also used as a non-G4 forming sequence control. 

**Figure 2:**
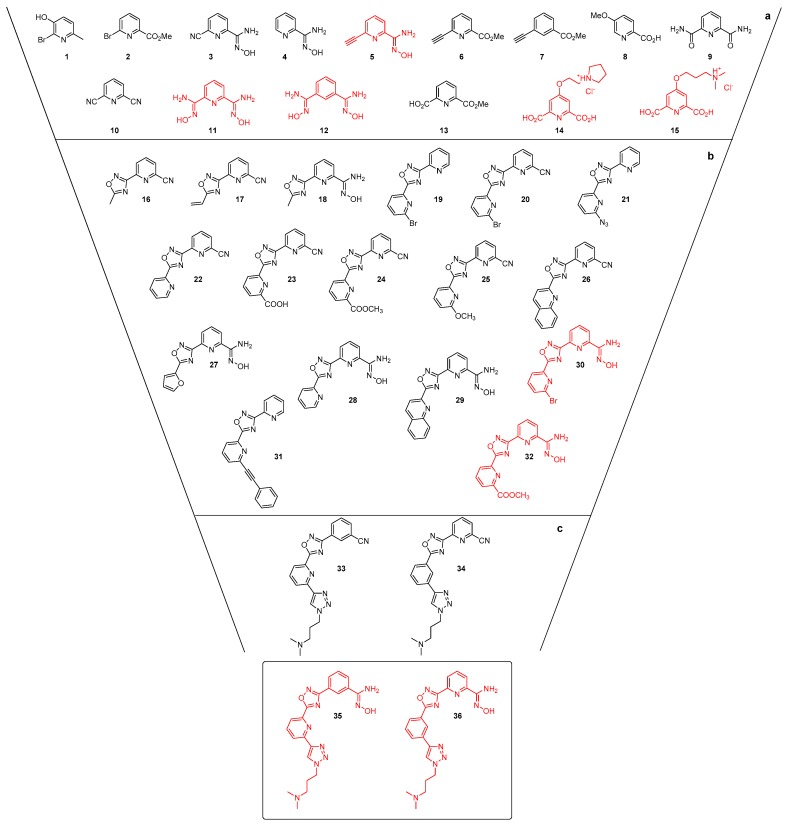
Fragment-based approach starting from (**a**) mono-aryl fragments, evolving trough (**b**) low level of complexity bi- and tri-aryl derivatives and (**c**) final tetra-heteroaryl hits.

Stabilization of the fragment library on these G4 structures was assessed by the HTS Fluorescence Resonance Energy Transfer (FRET) melting assay, using a 4000-fold excess of fragment vs. oligonucleotide. Similar compound/oligonucleotide ratio has been previously reported for different library investigation [38]. This preliminary evaluation allowed us to focus on a selected family of mono and bi functionalized single aromatic and hetero-aromatic rings (Figure 2a). Of the fifteen tested fragments belonging to this class, five displayed G4 stabilization in the range of 1.0–6.0 °C (Table 1, compounds **5**, **11**, **12**, **14** and **15**), without perturbation of the ds sequence except for **14**, the most potent one, which induced a moderate (2.5 °C) ds stabilization. 

**Scheme 1:**
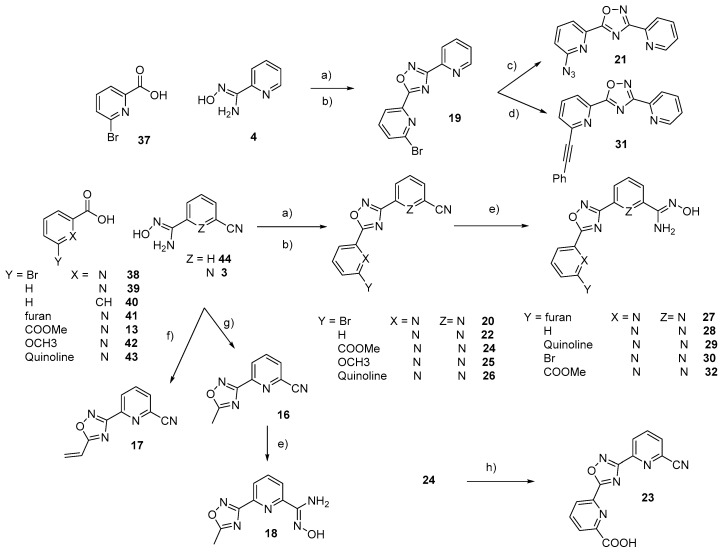
Synthetic protocol developed for the preparation of fragment compounds **18**–**36**. General condensation protocol: (a) 1,1′-carbonyldiimidazole (CDI), dry DMF, 16 h, r.t.; (b) 1,1′-carbonyldiimidazole (CDI), dry DMF, 6 h, 150 °C. (c) NaN_3_, DMF, 16 h, 100 °C; (d) Pd(PPh_3_)_2_Cl_2_, CuI, Phenylacetylene, THF/Et_3_N, 50 °C, 16 h, under argon atmosphere; (e) NH_2_OH/Na_2_CO_3_, H_2_O, 1 h, r.t.; (f) (i) Acroyl chloride, THF, 3 h, r.t., (ii) K_2_CO_3_, Dioxane, 6 h, 100 °C; (g) Ac_2_O, CHCl_3_, Et_3_N, 16 h, r.t.; (h) LiOH, THF/H_2_O, 4 h, r.t.

Target stabilization by fragments **14** and **15** is likely related to the presence of a protonated side chain under physiological conditions, which confers an additional electrostatic interaction with the DNA. The other three active fragments **5**, **11** and **12**, are more interesting from a chemical point of view due to the presence of a particular functional group, the amidoxime, which is sometimes considered a bioisostere for carboxylic group. Fragments **5**, **11** and **12** display an aromatic ring and one or two amidoximes group at 1,3 positions, directly linked to a pyrido or benzo central core (Scheme 1a).

This preliminarily SAR analysis indicated that, besides the presence of a water-soluble moiety, at least one unit of amidoxime improved ligand efficiency and potency, acting as a pharmacophore for LTR-III.

Amidoxime has been reported to play this role on different targets. In fact, several molecules containing the amidoxime group are biologically active: for example, they display hypotensive activities [39] and antibacterial and antitrypanocidic properties [40]. They were also found to generate NO in vivo, which had neuromodulatory and neurotransmitory effects. In addition, amidoxime and its derivatives are considered prodrugs of amidines in drug design [41]. Bearing both a hydroxyimino and an amino group at the same carbon atom, they are structurally close to amidines, amides and hydroxamic acids, chemical groups that are often used as mimetics of guanidine with consequent related pharmacological effects. 

### 2.2. Design, Synthesis and Analysis of the Polyheteroaryl Oxadiazole/Pyridine-Ligands

The most promising and versatile three hits (i.e., fragments **5**, **11** and **12**, Figure 2a) selected from the initial FRET screening were used as central scaffolds for the subsequent synthetic implementation (Figure 2b). In particular, starting from the mono-aromatic amidoxime core, we focused on the chain directionality of the polyaryl-structure, tuning the number of aromatic rings, adding different heteroaryl units and amino/cationic side chains. Moreover, to confirm the key role of the amidoxime, this moiety was replaced with a cyano-group in each structure. From a synthetic point of view, this was a straightforward task, as the cyano moiety is the precursor of the amidoxime.

Starting from the aromatic amidoxime central core (i.e., **3** or **44**, Scheme 1), we introduced different 3-substituted-1,2,4-oxadiazoles. The 3-methyl-1,2,4-oxadiazoles **16** and **18** (Scheme 1) were synthesized by an efficient cyclization protocol in the presence of acetic anhydride [42]. The formal arylation at the C3 of the 1,2,4-oxadiazole moiety was achieved in a single-step condensation protocol and subsequent cyclodehydration, using the amidoxime **3** or **44** and the carboxylic acid derivatives **13**, **38**–**43** (Scheme 1) as reactants. The condensation was optimised in the presence of 1,1’-carbonyldiimidazole as a coupling reagent to give the O-acylbenzamidoxime intermediates, which was detected by HPLC. The following one-pot cyclodehydration of the latter was achieved without the isolation of the intermediate, yielding the cyano-derivatives **20**, **22**, **24**–**26** in good yields. Finally, terminal amidoximes **27**–**30** and **32** were synthesised according to a standard protocol in the presence of hydroxylamine.

The newly synthesized fragments **16**–**32**, which ranged in molecular weights from 186 to 382 Da, were analysed by FRET melting assay. All the cyano and hydroxycarboxamidine-substituted new fragments showed stabilization of the G4 structures without significantly affecting the ds oligonucleotide (Table 1). The addition of a single 1,2,4-oxadiazole to a pyridine core, **16**, increased the stabilization to 3.6 °C. The binding properties of these implemented fragments are directly related to the number of aromatic/hetero-aromatic units in the structures (Table 1). The best three hits **27**, **30** and **32** were able to stabilize the G4s by 10.8–14.7 °C. These molecules were based on the 6-(1,2,4-oxadiazol-3-yl)pyridine-2-amidoxime template (see Scheme 1 for numbering), embedding an additional substituted-pyridine at C8. Fragments **30** and **32** were also analysed at 100-fold excess, in the presence of LTR-III and hTel G4s: in these conditions, **30** increased the melting temperature by 3.5 and 3.0 °C, respectively and **32** by 5.3 and 3.5 °C, respectively (Table 2). In addition, these two compounds did not show any stabilization on the ds sequence (Table 2). These data suggest a dose-dependent effect on G4 stabilization; however, no selectivity was observed between the two tested G4s. Finally, the ortho H substituted fragments **19**, **21** and **31** showed intense interference with the fluorescence signal in FRET analysis, therefore, T_m_ could not be calculated.

### 2.3. Design, Synthesis and Analysis of the Final Lead Compounds

**Scheme 2:**
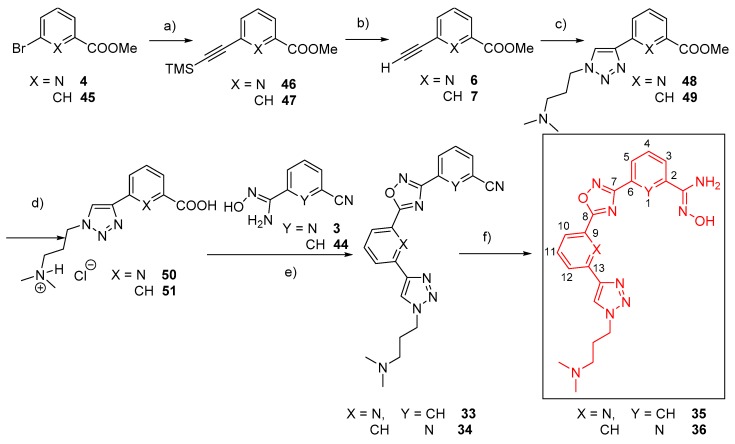
Synthetic protocol developed for the preparation of the final compounds **37**–**40**. (a) Pd(PPh_3_)_2_Cl_2_, CuI, PPh_3_, TMS-CCH, dry toluene/iPr_2_NH, 80 °C, 1 h; (b) K_2_CO_3_, MeOH, 2 h, r.t.; (c) CuSO_4_ 5H_2_O, NaAscorbate, H_2_O/tBuOH, 24 h, r.t.; (d) K_2_CO_3_, H_2_O/THF, 24 h, reflux.; (e) (i) 1,1′-carbonyldiimidazole (CDI), dry DMF, 16 h, r.t.; (ii) 1,1′-carbonyldiimidazole (CDI), dry DMF, 6 h, 150 °C; (f) NH_2_OH/Na_2_CO_3_, H_2_O, 1 h, r.t.

The third step (Figure 2c) of our implementation design aimed at the introduction of an additional heteroaromatic triazole moiety at C13 (see Scheme 1 and Scheme 2 for numbering) bearing a cationic side chain, to improve both the solubility and electrostatic binding to the target. The five-step synthetic protocol shown in Scheme 2 was optimized to prepare the final ligands **35**, **36** with excellent yields (overall yield >80%). Starting from the bromo-derivatives **4** and **45**, a Sonogashira cross-coupling was performed with ethynyltrimethylsilane. The subsequent deprotection of the trimethylsilyl (TMS) group upon basic methanolysis yielded the terminal alkynes **6** and **7** in high yields (90%) and mild conditions. The copper(I)-catalysed alkyne-azide cycloaddition (CuAAC) reaction between 3-azido-*N*,*N*-dimethylpropan-1-amine and the terminal alkynes **6** or **7** was carried out in aqueous *tert*-butanol to dissolve all the reactants and implement reaction yields affording **48** and **49**, which were used for the next hydrolytic step without purification. Both the resulting carboxylic acid derivatives **50** and **51** were reacted with the amidoximes **3** and **44** according to the previously described protocol. The two cyano derivatives **33** and **34** were converted into the final ligands **35** and **36** with two equivalents of hydroxylamine in aqueous ethanol solution (Scheme 2 and Figure 2c). In order to highlight the role of amidoxime, a key binding moiety, we decided to compare **35** and **36** to their cyano analogues **33** and **34** (Figure 2c), which maintain the identical scaffold.

**Figure 3:**
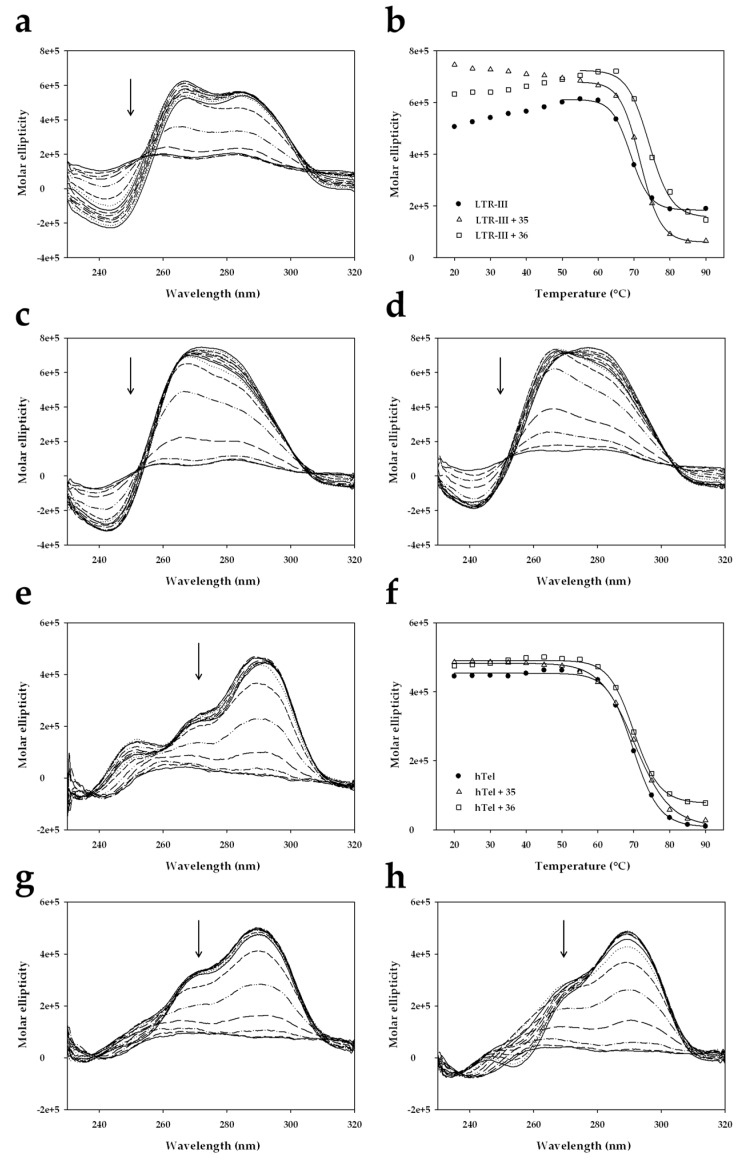
CD thermal unfolding analysis. CD of LTR-III G4 (1.5 μM) at K^+^ 100 mM in the absence (**a**) and in the presence of 10-fold excess of **35** (**c**) and **36** (**d**) (15 μM). CD of hTel G4 (1.5 μM) at K^+^ 100 mM in the absence (**e**) and in the presence of 10-fold excess of **35** (**g**) and **36** (**h**) (15 μM). CD spectra variation is shown as a function of the wavelength; arrows indicate the spectral change from low to high temperatures. The molar ellipticity at the peak wavelength (265 nm for LTR-III G4 (**b**) and 290 nm for hTel G4 (**f**)) is shown as a function of the temperature.

The four new compounds **33**–**36** (MW 400–433 Da) showed improved stabilization properties: ΔT_m_ at 100-fold excess of compound was 8.9–15.5 °C (Table 2) on G4s and, at 4000-fold excess, was higher than 20 °C, the maximum level measurable in this condition (Table 1). However, fragments **33** and **34** greatly stabilized also dsDNA (ΔT_m_ > 28.0 °C at 4000-fold excess of compound, Table 1). For this reason, only fragments **35** and **36** were further investigated. In particular, the ability of **35** and **36** to stabilize LTR-III and hTel G4s was confirmed by circular dichroism (CD). At 10-fold excess of compound vs. oligonucleotide, the highest ratio achievable in CD conditions, **35** and **36** stabilized LTR-III of 1.7 ± 0.5 °C (Figure 3c) and 5.1 ± 0.7 °C (Figure 3d), respectively, also inducing a slight conformational change. The same compounds induced lower stabilization on hTel G4, that is, ΔT_m_ = 0.2 ± 0.1 °C (Figure 3g) and 0.5 ± 0.2 °C (Figure 3h), respectively.

**Figure 4:**
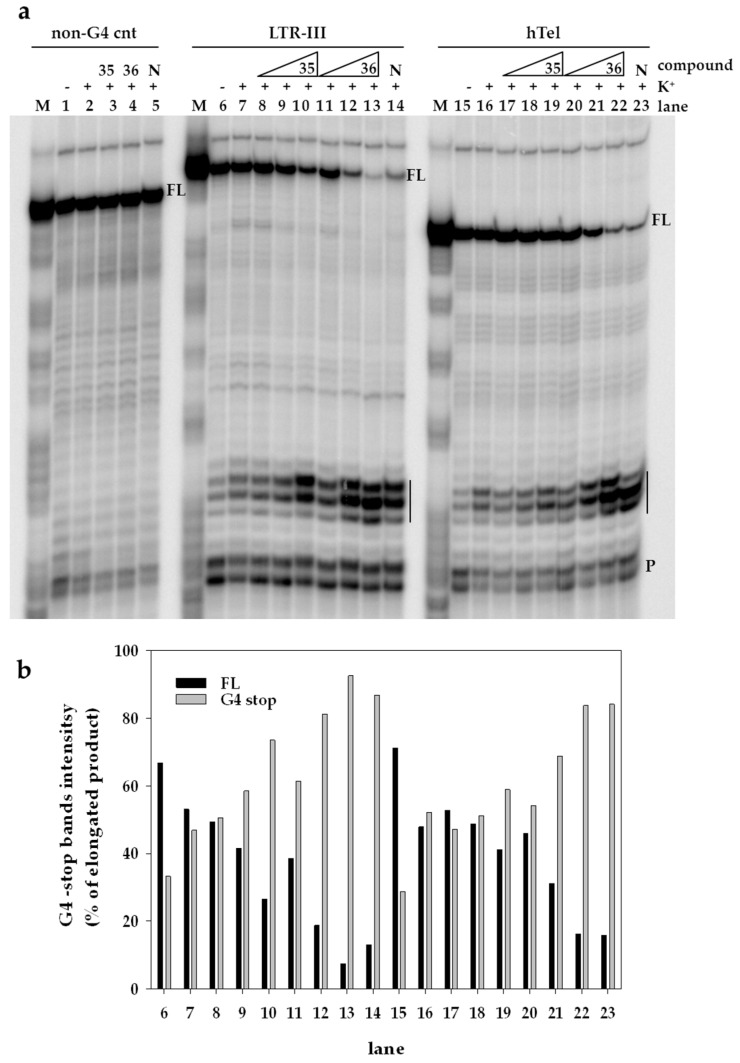
Image of a typical Taq polymerase stop assay. (**a**) LTR-III and hTel templates were amplified by Taq polymerase in the absence (lanes 6 and 15) and presence of 100 mM K^+^, alone (lanes 7 and 16) or with increasing amounts (50, 100 and 200 nM) of **35** (lanes 8–10 and 17–19) or **36** (lanes 11–13 and 20–22). NDI (N) 200 nM (lanes 14 and 23) was used as control for a non-selective G4-ligand. A template (non-G4 cnt) made of a scrambled sequence unable to fold into G4 was also used as internal control in the absence (lane 1) and presence of 100 mM K^+^, alone (lane 2) or with 200 nM of **35** (lane 3), **36** (lane 4), N (lane 5). Lane P: unreacted labelled primer. Lane M: ladder of markers obtained by the Maxam and Gilbert sequencing carried out on the amplified strand complementary to the template strand. Vertical bars indicate G4-specific Taq polymerase stop sites. (**b**) Quantification of lanes 6–23 shown in panel a. Quantification of stop bands corresponding to G4 and of the full-length amplification product (FL) is shown.

The Taq polymerase stop assay was next performed to check if these two hits were able to inhibit polymerase progression at the G4 site. Extended LTR-III and hTel G4 forming sequences were used, containing additional flanking bases at the 3′-end: a primer annealing sequence and a 5-T linker region to separate the annealing sequence from the first G of the G4 tract. In the absence of compounds and in the presence of 100 mM K^+^, both G4 forming sequences stopped the polymerase at the most 3′-end G-rich region, that is, the first G-rich region encountered by the enzyme, indicating that K^+^ stimulates G4 folding (Figure 4a, lanes 7 and 16). Upon addition of increasing amounts (50–200 nM) of **35** and **36**, the intensity of the stop bands highly increased in all templates (Figure 4a, lanes 8–13 and 17–22) along with considerable reduction of the full-length amplicons, thus corroborating the effective stabilization of the G4s by both compounds at nM concentrations. In contrast, all tested fragments had no effect on a DNA template unable to fold into G4 (Figure 4a, lanes 3–4), indicating that the observed polymerase inhibition was G4-dependent. In accordance to the FRET melting data, **36** displayed a higher G4 stabilizing effect on both templates with respect to **35**. In addition, **36** was mildly selective towards LTR-III vs. hTel (see stop bands quantification in Figure 4b). A naphthalene diimide (NDI) ligand [43] was used as internal control, as it is a well-characterized G4 ligand that displays no selectivity between LTR-III and hTel G4s (Figure 4a, lanes 14 and 23).

**Figure 5:**
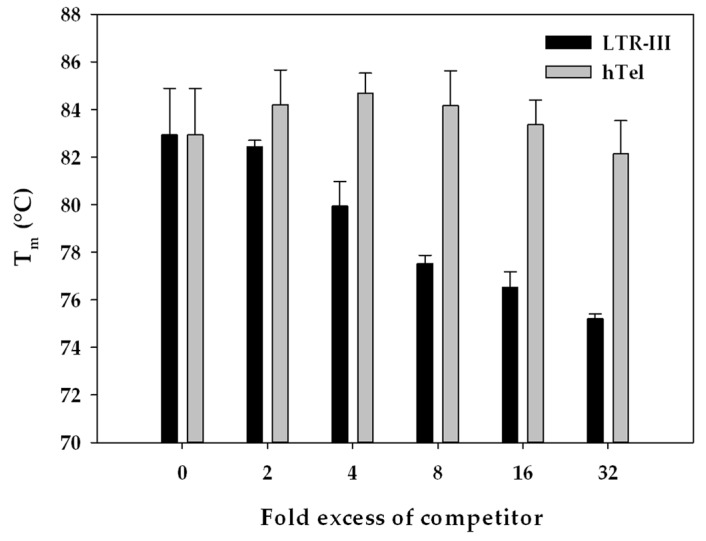
Competition analysis by FRET assay. 5′-FAM- and 3′-TAMRA-labeled LTR-III G4 (0.25 μM) was mixed with increasing concentrations (0–32-fold excess) of unlabelled competitor LTR-III or hTel and a constant amount (25 μM) of **36**.

To validate the preferential binding of **36** to LTR-III with respect to hTel, we performed FRET competition assay. 5′-FAM- and 3′-TAMRA-labeled LTR-III was mixed with increasing concentrations of the unlabelled competitor, LTR-III or hTel (Figure 5) and a constant amount of **36**. Unlabelled LTR-III and hTel G4 sequences displayed T_m_ of 68.6 ± 0.5 °C and 68.7 ± 0.1 °C respectively, sufficiently similar to allow a meaningful comparison. No significant variation of T_m_ was observed with hTel as the unlabelled competitor, indicating that the latter did not compete with the labelled LTR-III for the binding to **36**. On the contrary, a dose dependent decrease of T_m_ was detected in the presence of LTR-III unlabelled competitor, suggesting that **36** bound both labelled and unlabelled oligonucleotides. These data demonstrate that, in the presence of both G4s, **36** preferentially binds to LTR-III over hTel.

## 3. Conclusions

A FBDD approach was successfully applied to develop compounds against a target G4. An unanticipated moiety, the amidoxime, was found to consistently enhance compound activity. Our data indicate that FBDD may be a valuable approach to generate new pharmacophores that specifically recognize G4 nucleic acid structures. Considering that one of the main obstacles in the development of G4 ligands is their size and consequent poor pharmacokinetics properties, the presented approach, which adds up fragments that singly bind the target, indicates a possible way to develop compounds with smaller size and more drug-like properties.

## 4. Materials and Methods

### 4.1. General Information

All chemicals and solvents were purchased from Sigma Aldrich (Milan, Italy) and used without further purification. TLC analysis was carried out on silica gel (Merck 60F 254, Milan, Italy) with visualization at 254 and 366 nm. Flash chromatography was performed with silica gel 60 (40–63 µm, Merck, Milan, Italy). All anhydrous reactions were carried out under positive pressure of nitrogen or argon. Elemental analysis was provided by a Carlo Erba CHN analyser (Milan, Italy). All ^1^H-NMR and ^13^C-NMR spectra were recorded on a Bruker Advance 300 MHz spectrometer (Billerica, MA, USA) using deuterated solvents and TMS as internal standard. The spectra are reported in ppm and referenced to deuterated DMSO (2.49 ppm for ^1^H, 39.5 ppm for ^13^C) or deuterated chloroform (7.26 ppm for ^1^H, 77 ppm for ^13^C). The following abbreviations are used: singlet (s), doublet (d), triplet (t) and multiplet (m). Naked and modified oligonucleotide sequences were purchased from Sigma Aldrich (Milan, Italy). The NMR Figures were provided in Supplementary Materials.

### 4.2. Synthetic Methods

#### 4.2.1. Synthesis of the Fragment Family Shown in Figure 2a

Compounds **1**, **2**, **4**, **6**, **7**, **8**, **10** and **13** were purchased from commercial sources Merck-Sigma Aldrich, while the fragments **3**, **9**, **11**, **12**, **14** and **15** have been synthesized according to the published procedures [42,44,45,46].

*(Z)-6-Ethynyl-N′-hydroxypicolinimidamide,* fragment **5**: An aqueous solution (45 mL) of hydroxylamine hydrochloride (0.11 g, 1.58 × 10^−3^ mol) and Na_2_CO_3_ (0.167 g, 1.58 × 10^−3^ mol), was added dropwise in 30 min at r.t., to a solution of 6-ethynyl-2-pyridinecarbonitrile (0.166 g, 1.29 × 10^−3^ mol) in ethanol (95 mL). The resulting mixture was stirred at room temperature for 2 h. Then the organic solvent was removed under vacuum in order to induce the precipitation of the product. The light brown solid was filtered and dried (0.149 g, yield 71%). ^1^H-NMR (300 MHz, DMSO-*d*_6_): δ = 10.04 (s, 1H), 7.85–7.83 (m, 2H), 7.57–7.59 (m, 1H), 5.79 (s, 2H), 4.4 (s, 1H). ^13^C-NMR (75 MHz, DMSO-*d*_6_): δ = 150.5, 148.9, 140.3, 137.3, 119.5, 82.8, 80.6; elemental analysis calcd (%) for C_8_H_7_N_3_O: C, 59.62; H, 4.38; N, 26.07; found C, 59.67; H, 4.37; N, 26.01.

#### 4.2.2. Synthesis of the Fragment Family Shown in Figure 2b

*6-(5-Methyl-1,2,4-oxadiazol-3-yl)picolinonitrile*, fragment **16**: 0.3 g of (*Z*)-6-cyano-*N′*-hydroxypicolinimidamide (1.85 × 10^−3^ mol) was solved in 15 mL of chloroform and treated with 0.21 mL of Ac_2_O (2.22 × 10^−3^ mol) and 0.3 mL of Et_3_N. The resulting mixture was stirred at room temperature overnight. The solvent was removed under pressure and the (Z)-6-cyano-2-*N*-acetoxy-amidoxime-pyridine obtained was directly treat with 5 mL of glacial acetic acid refluxing the solution for 4 h. The neutralization of the reaction mixture with a saturated solution of sodium bicarbonate induce the precipitation of the product. The white solid was filtered and dried under vacuum (0.225 g, yield 65%). ^1^H-NMR (300 MHz, CDCl_3_, 25 °C, TMS): δ = 8.35 (d, 3*J*(H, H) = 7.8 Hz, 1 H), 8.05 (t, 3*J*(H, H) = 7.8 Hz, 1H), 7.86 (d, 3*J*(H, H) = 7.8 Hz, 1H), 2.74 (s, 3H). ^13^C-NMR (75 MHz, CDCl_3_, 25 °C, TMS): δ = 179.8, 168.7, 149.7, 140.1, 136.3, 131.7, 127.8, 118.1, 14.2; elemental analysis calcd (%) for C_9_H_6_N_4_O: C, 58.06; H, 3.25; N, 30.09; found C, 58.04; H, 3.27; N, 30.10. 

*6-(5-Vinyl-1,2,4-oxadiazol-3-yl)picolinonitrile*, fragment **17**: acroyl chloride (0.12 mL, 1.48 × 10^−3^ mol) was added to a solution of(*Z*)-6-cyano-*N′*-hydroxypicolinimidamide (0.2 g, 3.7 × 10^−3^ mol) in 15 mL of chloroform. The reaction mixture was stirred at room temperature for 3 h, then quenched with a saturated solution of NaHCO_3_ and extracted with chloroform (3 × 20 mL). The organic phase was dried under vacuum and the intermediate obtained was directly used for the cyclization step. The crude was dissolved in 25 mL of 1,4-dioxane, treat with K_2_CO_3_ (0.335 g, 2.4 × 10^−3^ mol) and heated at 100 °C for 6 h. The reaction mixture was cooled to r.t. and the solvent was dried under vacuum. The crude solid was treat with chloroform in order to dissolve only the product. The organic phase was then dried under vacuum (white solid, 0.292 g, yield 80%). ^1^H-NMR (300 MHz, CDCl_3_, 25 °C, TMS): δ = 8.5 (dd, 3*J*(H, H) = 8.0 Hz; 3*J*(H, H) = 1.1 Hz, 1H), 8.16 (t, 3*J*(H, H) = 7.9 Hz, 1H), 7.97 (dd, 3*J*(H, H) = 7.9 Hz; 3*J*(H, H) = 1.1 Hz, 1H), 6.95 (dd, 3*J*(H, H) = 17.7 Hz; 3*J*(H, H) = 10.7 Hz, 1H), 6.81 (dd, 3*J*(H, H) = 17.7 Hz; 3*J*(H, H) = 1.1 Hz, 1H), 6.21 (dd, 3*J*(H, H) = 10.7 Hz; 3*J*(H, H) = 1.1 Hz, 1H). ^13^C-NMR (75 MHz, CDCl_3_, 25 °C, TMS): δ = 175.6, 167.1, 147.9, 138.3, 134.5, 130.0, 129.9, 126.1, 120.0, 116.3; elemental analysis calcd (%) for C_10_H_6_N_4_O: C, 60.60; H, 3.05; N, 28.27; found C, 60.62; H, 3.01; N, 28.27.

General procedure A for the synthesis of the amidoxime derivatives **18**, **27**, **28**, **29**, **32**, **35** and **36**. A mixture of hydroxylamine hydrochloride (0.68 mmol) and of Na_2_CO_3_ (0.34 mmol) in water (10 mL) was added dropwise in 45 min to a solution containing the nitrile derivatives (0.33 mmol) in EtOH (30 mL). The reaction mixture was stirred at r.t. for 1 h. The precipitated white solid was filtered and dried. All the carbonitrile fragment were converted in the corresponding amidoxime derivatives with high yields (Z)-*N′*-hydroxy-6-(5-methyl-1,2,4-oxadiazol-3-yl)picolinimidamide, fragment **18**: white solid, yield 89%. ^1^H-NMR (300 MHz, DMSO-*d*_6_): δ = 10.13 (s, 1 H), 8.06–8.02 (m, 3H), 5.87 (s, 2H), 2.71 (s, 3H). ^13^C-NMR (75 MHz, DMSO-*d*_6_): δ = 177.9, 167.3, 150.5, 148.7, 144.5, 138.2, 123.3, 121.5, 12.1; elemental analysis calcd (%) for C_9_H_9_N_5_O_2_: C, 49.31; H, 4.14; N, 31.95; found C, 49.38; H, 4.12; N, 31.91.

General cyclization protocol B for the synthesis of the 1,2,4-oxadiazole derivatives **19**, **20**, **22**, **24**, **25**, **26**, **33** and **34**. The opportune carboxylic acid (1.46 mmol) and 1,1′-carbonyldiimidazole (CDI, 1.46 mmol) were dissolved in DMF (10 mL) and stirred for 30 min at r.t.. After this period, the correspondent amidoxime derivatives (1.46 mmol) was added and the reaction mixture was stirred at r.t. overnight. CDI (1.46 mmol) was further added and the reaction mixture was heated at 150 °C for 6 h. After cooling down, the resulting solution was poured into water to induce the precipitation of a solid, which was filtered and characterised as pure product. 5-(6-Bromopyridin-2-yl)-3-(pyridin-2-yl)-1,2,4-oxadiazole, fragment **19**: white solid, yield 53%. ^1^H-NMR (300 MHz, DMSO-*d*_6_): δ = 8.81 (d, 3*J*(H, H) = 4.0 Hz; 1H), 8.38 (dd, 3*J*(H, H) = 7.4 Hz; 3*J*(H, H) = 0.9 Hz, 1H), 8.2 (d, 3*J*(H, H) = 7.4 Hz; 1H), 8.12–8.00 (m, 3H), 7.68–7.63 (m, 1H). ^13^C-NMR (75 MHz, DMSO-*d*_6_): δ = 173.3, 168.5, 150.4, 145.4, 143.3, 141.8, 141.3, 137.8, 132.1, 126.4, 124.0, 123.5; elemental analysis calcd (%) for C_12_H_7_BrN_4_O: C, 47.55; H, 2.33; N, 18.48; found C, 47.58; H, 2.31; N, 18.45.

*6-(5-(6-Bromopyridin-2-yl)-1,2,4-oxadiazol-3-yl)picolinonitrile*, fragment **20**: general method B. White solid, yield 57%. ^1^H-NMR (300 MHz, DMSO-*d*_6_): δ = 8.48 (d, 3*J*(H, H) = 9.0 Hz; 1 H), 8.38 (t, 3*J*(H, H) = 7.4 Hz, 1H), 8.32 (d, 3*J*(H, H) = 9 Hz; 1H), 8.23 (d, 3*J*(H, H) = 7.4 Hz; 1H), 8.08 (t, 3*J*(H, H) = 7.7 Hz, 1 H), 7.99 (d, 3*J*(H, H) = 7.7 Hz; 1H). ^13^C-NMR (75 MHz, DMSO-*d*_6_): δ = 173.8, 167.5, 146.9, 143.2, 141.9, 141.1, 139.9, 133.4, 132.1, 130.9, 127.1, 124.1, 116.7; elemental analysis calcd (%) for C_13_H_6_BrN_5_O: C, 47.59; H, 1.84; N, 21.34; found C, 48.01; H, 1.83; N, 21.36.

*5-(6-Azidopyridin-2-yl)-3-(pyridin-2-yl)-1,2,4-oxadiazole*, fragment **21**: 0.108 g of sodium azide (1.66 × 10^−3^ mol) was added to a solution of (**19**) (0.05 g 1.66 × 10^−3^ mol) in 2 mL of dimethylformammide. The reaction mixture was heated at 100 °C and stirred overnight. The solvent was removed under pressure, the crude product was washed with water and filtered in order to obtain a pure white solid (0.038 g, yield 87%). ^1^H-NMR (300 MHz, CDCl_3_, 25 °C, TMS): δ = 8.87 (d, 3*J*(H, H) = 4.8 Hz, 1H), 8.28 (d, 3*J*(H, H) = 7.9 Hz, 1H), 8.19 (dd, 3*J*(H, H) = 7.6 Hz; 3*J*(H, H) = 0.7 Hz, 1H), 7.94–7.85 (m, 2H), 7.52–7.47 (m, 1H), 7.01 (dd, 3*J*(H, H) = 8.0 Hz; 3*J*(H, H) = 0.7 Hz, 1H). ^13^C-NMR (75 MHz, CDCl_3_, 25 °C, TMS): δ = 174.3, 168.8, 155.5, 150.4, 146.0, 142.4, 139.7, 137.0, 126.1, 123.7, 120.7, 117.6; elemental analysis calcd (%) for C_12_H_7_N_7_O: C, 54.34; H, 2.66; N, 36.97; found C, 54.32; H, 2.65; N, 37.01. 

*6-(5-(Pyridin-2-yl)-1,2,4-oxadiazol-3-yl)picolinonitrile*, fragment **22**: general method B. White solid, yield 45%. ^1^H-NMR (300 MHz, DMSO-*d*_6_): δ = 8.9 (d, 3*J*(H, H) = 4.2 Hz; 1H), 8.5 (dd, 3*J*(H, H) = 7.7 Hz; 3*J*(H, H) = 1.1 Hz, 1H), 8.4–8.27 (m, 3H), 8.15 (dt, 3*J*(H, H) = 7.7 Hz; 3*J*(H, H) = 1.6 Hz 1H), 7.79–7.74 (m, 1H). ^13^C-NMR (75 MHz, DMSO-*d*_6_): δ = 171.1, 167.3, 150.7, 146.9, 142.5, 140.0, 138.2, 133.3, 131.1, 127.7, 127.1, 124.7, 116.9; elemental analysis calcd (%) for C_13_H_7_N_5_O: C, 62.65; H, 2.83; N, 28.10; found C, 62.63; H, 2.81; N, 28.14.

*6-(3-(6-Cyanopyridin-2-yl)-1,2,4-oxadiazol-5-yl)picolinic acid^.^2HCl*, fragment **23**: to a solution of fragment **24** (0.100 g, 3.25 × 10^−4^ mol) in THF/H_2_O (2:1, 6 mL), were added 0.07 g of LiOH H_2_O (1.62 × 10^−3^ mol). The reaction mixture was stirred for 4 h at r.t., after this period, the solution was acidified with chloridric acid (1 mL) and the organic solvent removed under pressure. The product precipitate was filtered and washed with water in order to obtain a pure white solid (0.087 g, yield 91%). ^1^H-NMR (300 MHz, DMSO-*d*_6_): δ = 8.6–8.5 (m, 1H), 8.41–8.35 (m, 4H), 8.32–8.26 (m, 1H), 8.0 (bs, 1H), 7.9 (bs, 1H). ^13^C-NMR (75 MHz, DMSO-*d*_6_): δ = 174.6, 167.9, 165.3, 151.1, 149.5, 146.8, 144.4, 140.1, 133.3, 128.0, 125.9, 116.8; elemental analysis calcd (%) for C_14_H_9_Cl_2_N_5_O_3_: C, 45.92; H, 2.48; N, 19.13; found C, 44.88; H, 2.46; N, 19.16.

*Methyl 6-(3-(6-cyanopyridin-2-yl)-1,2,4-oxadiazol-5-yl)picolinate*, fragment **24**: general method B. White solid, yield 46%. ^1^H-NMR (300 MHz, DMSO-*d*_6_): δ = 8.60 (dd, 3*J*(H, H) = 6.0 Hz; 3*J*(H, H) = 2.9 Hz; 1H), 8.51 (dd, 3*J*(H, H) = 7.6 Hz; 3*J*(H, H) = 1.0 Hz; 1H), 8.38–8.29 (m, 4H), 3.98 (s, 3H). ^13^C-NMR (75 MHz, DMSO-*d*_6_): δ = 174.4, 167.3, 164.3, 148.3, 146.7, 142.7, 140.1, 140.0, 133.3, 131.1, 128.2, 127.8, 127.1 116.9, 52.8; elemental analysis calcd (%) for C_15_H_9_N_5_O_3_: C, 58.63; H, 2.95; N, 22.79; found C, 58.69; H, 2.93; N, 22.82.

*6-(5-(6-Methoxypyridin-2-yl)-1,2,4-oxadiazol-3-yl)picolinonitrile^.^2HCl*, fragment **25**: general method B. White solid, yield 19%. ^1^H-NMR (300 MHz, DMSO-*d*_6_): δ = 8.55 (d, 3*J*(H, H) = 3.9 Hz; 1H), 8.37–8.32 (m, 2H), 8.25–8.22 (m, 2H), 7.96 (bs, 1H), 7.87 (bs, 1H), 7.67 (dd, 3*J*(H, H) = 8.8 Hz; 3*J*(H, H) = 2.8 Hz; 1H), 3.96 (s, 3H). Elemental analysis calcd (%) for C_14_H_11_Cl_2_N_5_O_2_: C, 47.75; H, 3.15; N, 19.89; found C, 47.72; H, 3.18; N, 19.85.

*6-(5-(Quinolin-2-yl)-1,2,4-oxadiazol-3-yl)picolinonitrile*, fragment **26**: general method B. White solid, yield 60%. ^1^H-NMR (300 MHz, DMSO-*d*_6_): δ = 8.73 (d, 3*J*(H, H) = 8.4 Hz; 1H), 8.53 (d, 3*J*(H, H) = 7.8 Hz, 1H), 8.43 (d, 3*J*(H, H) = 7.8 Hz; 1H), 8.36 (t, 3*J*(H, H) = 7.8 Hz; 1H), 8.29–8.26 (m, 2H), 8.17 (d, 3*J*(H, H) = 8.0 Hz; 1H), 7.96 (t, 3*J*(H, H) = 7.1 Hz; 1H), 7.82 (t, 3*J*(H, H) = 7.4 Hz; 1H). ^13^C-NMR (75 MHz, DMSO-*d6*): δ = 175.2, 167.5, 147.4, 147.0, 142.6, 139.9, 138.4, 133.4, 131.2, 130.1, 129.7, 129.1, 128.9, 127.1, 120.6, 116.8; elemental analysis calcd (%) for C_17_H_9_N_5_O: C, 68.22; H, 3.03; N, 23.40; found C, 68.18; H, 3.05; N, 23.38.

*(Z)-6-(5-(Furan-2-yl)-1,2,4-oxadiazol-3-yl)-N′-hydroxypicolinimidamide*, fragment **27**: general method A. White solid, yield 90%. ^1^H-NMR (300 MHz, DMSO-*d*_6_): δ = 10.15 (s, 1H), 8.20 (d, 3*J*(H, H) = 0.7 Hz; 1H), 8.14 (q, 3*J*(H, H) = 4.4 Hz, 1H), 8.11–7.96 (m, 2H), 7.73 (d, 3*J*(H, H) = 7.8 Hz; 1H), 6.91–6.74 (m, 1H), 5.89 (s, 2H). ^13^C-NMR (75 MHz, DMSO-*d*_6_): δ = 167.7, 167.6, 150.6, 148.8, 148.6, 144.1, 138.9, 138.3, 123.7, 121.8, 117.9, 113.2; elemental analysis calcd (%) for C_12_H_9_N_5_O_3_: C, 53.14; H, 3.34; N, 25.82; found C, 53.20; H, 3.36; N, 25.80.

*(Z)-N′-Hydroxy-6-(5-(pyridin-2-yl)-1,2,4-oxadiazol-3-yl)picolinimidamide*, fragment **28**: general method A. White solid, yield 86%. ^1^H-NMR (300 MHz, DMSO-*d*_6_): δ = 10.26 (s, 1H), 8.99–8.97 (m, 1H), 8.48 (d, 3*J*(H, H) = 6.8 Hz; 1H), 8.33–8.23 (m, 2H), 8.20 (d, 3*J*(H, H) = 1.28 Hz; 1H), 8.18 (s, 1H), 7.89–7.84 (m, 1H), 6.02 (s, 2H). ^13^C-NMR (75 MHz, DMSO-*d*_6_): δ = 174.7, 168.1, 150.6, 148.8, 144.3, 142.7, 140.1, 138.3, 138.2, 127.6, 124.6, 123.6, 121.8; elemental analysis calcd (%) for C_13_H_10_N_6_O_2_: C, 55.32; H, 3.57; N, 29.77; found C, 55.28; H, 3.56; N, 29.82.

*(Z)-N′-Hydroxy-6-(5-(quinolin-2-yl)-1,2,4-oxadiazol-3-yl)picolinimidamide*, fragment **29**: general method A. White solid, yield 92%. ^1^H-NMR (300 MHz, DMSO-*d*_6_): δ = 10.09 (s, 1H), 8.24 (d, 3*J*(H, H) = 8.5 Hz; 1H), 8.45 (d, 3*J*(H, H) = 8.5 Hz, 1H), 8.27–8.25 (m, 2H), 8.18 (d, 3*J*(H, H) = 8.2 Hz; 1H), 8.11 (d, 3*J*(H, H) = 4.1 Hz; 2H), 7.96 (dt, 3*J*(H, H) = 8.0 Hz, 3*J*(H, H) = 1.4 Hz, 1H), 7.82 (dt, 3*J*(H, H) = 8.0 Hz, 3*J*(H, H) = 1.4 Hz, 1H), 5.89 (s, 2H). ^13^C-NMR (75 MHz, DMSO-*d*_6_): δ = 174.8, 168.3, 150.8, 148.9, 147.3, 144.3, 142.8, 138.4, 138.3, 131.2, 129.7, 129.1, 128.9, 128.3, 123.7, 121.8, 120.6; elemental analysis calcd (%) for C_17_H_12_N_6_O_2_: C, 61.44; H, 3.64; N, 25.29; found C, 61.49; H, 3.62; N, 25.25.

Compound **30** has been synthesized according to the published procedures [42].

*(Z)-N′-Hydroxy-6-(5-(6-(phenylethynyl)pyridin-2-yl)-1,2,4-oxadiazol-3-yl)picolinimidamide*, fragment **31**: Pd(Ph_3_P)_2_Cl_2_ (0.005 g, 7.1 × 10^−6^ mol) and CuI (0.0006 g, 3.15 × 10^−6^ mol) were added to a suspension of **19** (0.1 g 3.3 × 10^−4^ mol) in 5 mL of THF/Et_3_N (1:1). The solution was stirred for fiftheen min at r.t.. After the addition of ethynylbenzene (0.128 mL, 1.16 × 10^−3^ mol), the mixture was stirred overnight, at 50 °C, under nitrogen atmosphere. After this period, the solvent was removed under vacuum. The resulting crude was treat with 20 mL of water and then extracted with three portions of chloroform (3 × 20 mL). The organic phase was dried on Na_2_SO_4_, filtered and removed under reduced pressure. The crude product was purified by column chromatography and the product was obtained as a yellow solid (0.067 g, yield 63%). ^1^H-NMR (300 MHz, CDCl_3_, 25 °C, TMS): δ = 8.85 (d, 3*J*(H, H) = 4.0 Hz, 1H), 8.37 (d, 3*J*(H, H) = 7.0 Hz, 1H), 8.29 (d, 3*J*(H, H) = 7.8 Hz, 1H), 7.95 (d, 3*J*(H, H) = 7.8 Hz, 1H), 7.93–7.87 (m, 1H), 7.75 (dd, 3*J*(H, H) = 7.8 Hz, 3*J*(H, H) = 0.8 Hz, 1H), 7.66–7.63 (m, 2H), 7.49–7.45 (m, 1H), 7.42–7.37 (m, 3H). ^13^C-NMR (75 MHz, CDCl_3_, 25 °C, TMS): δ = 174.4, 168.8, 150.4, 146.0, 144.4, 143.7, 137.5, 137.9, 132.1, 130.1, 129.3, 128.3, 125.6, 123.4, 123.2, 121.6, 91.0, 87.6; elemental analysis calcd (%) for C_21_H_14_N_6_O_2_: C, 65.96; H, 3.69; N, 21.98; found C, 66.00; H, 3.66; N, 21.97.

*Methyl (Z)-6-(3-(6-(N′-hydroxycarbamimidoyl)pyridin-2-yl)-1,2,4-oxadiazol-5-yl)picolinate*, fragment **32**: general method A. White solid, yield 88%. ^1^H-NMR (300 MHz, DMSO-*d*_6_): δ = 10.16 (bs, 1H), 8.60 (dd, 3*J*(H, H) = 6.2 Hz; 3*J*(H, H) = 2.7 Hz; 1H), 8.38–8.32 (m, 2H), 8.25–8.21 (m, 1H), 8.11–8.08 (m, 2H), 5.91 (s, 2H), 3.97 (s, 3H). ^13^C-NMR (75 MHz, DMSO-*d*_6_): δ = 174.1, 168.2, 164.4, 150.7, 148.8, 148.3, 144.2, 142.9,139.9, 138.3, 128.1, 127.8, 123.7, 121.9, 52.8; elemental analysis calcd (%) for C_15_H_12_N_6_O_4_: C, 52.94; H, 3.55; N, 24.70; found C, 52.91; H, 3.54; N, 24.74.

#### 4.2.3. Synthesis of the Fragment Family Shown in Figure 2c

Synthesis of intermediate **48**–**49**: The alkyne (**6** or **7**, 1.24 mmol) was dissolved in tert-butanol (10 mL) and added to a water solution (10 ml) of *N*,*N*-Dimethyl-*N*-(3-azidopropyl)amine hydrochloride (226 mg, 1.37 mmol), copper (II) sulphate pentahydrate (31 mg, 10% mol) and sodium ascorbate (246 mg, 1.24 mmol). The resulting mixture was stirred for 24 h, at r.t. The organic solvent was removed under vacuum and the residue was diluted with saturated NaHCO_3_ (10 mL) and extracted with DCM (3 × 20 mL). The combined organic phases were dried over Na_2_SO_4_ and the solvent was removed under reduced pressure. The crude was purified by flash chromatography (CHCl_3_/MeOH 95:5).

*Methyl 6-(1-(3-(dimethylamino)propyl)-1H-1,2,3-triazol-4-yl)picolinate* (**48**): colourless liquid, yield = 90%. ^1^H-NMR (300 MHz, CDCl_3_, 25 °C, TMS) δ (ppm) 8.39 (d, *J* = 7.9 Hz, 1H), 8.36 (s, 1H), 8.06 (d, *J* = 7.7 Hz, 1H), 7.95 (t, *J* = 7.8 Hz, 1H), 4.53 (t, *J* = 7.0 Hz, 2H), 4.02 (s, 3H), 2.34 (t, *J* = 6.8 Hz, 2H), 2.25 (s, 6H), 2.14 (quint, *J* = 6.9 Hz, 2H). ^13^C-NMR (75 MHz, CDCl_3_, 25 °C, TMS) δ (ppm) 165.4, 150.7, 147.5, 147.3, 137.7, 123.9, 123.2, 122.9, 55.7, 52.6, 48.2, 45.2, 28.0.

*Methyl 3-(1-(3-(dimethylamino)propyl)-1H-1,2,3-triazol-4-yl)benzoate* (**49**): colourless liquid, yield = 95%. ^1^H-NMR (300 MHz, CDCl_3_, 25 °C, TMS) δ (ppm) 8.42 (s, 1H), 8.11 (d, *J* = 7.8 Hz 1H), 7.99 (d, *J* = 7.8 Hz, 1H), 7.89 (s, 1H), 7.51 (t, *J* = 7.8 Hz, 1H), 4.49 (t, *J* = 6.9 Hz, 2H), 3.94 (s, 3H), 2.31 (t, *J* = 6.7 Hz, 2H), 2.24 (s, 6H), 2.11 (quint, *J* = 6.8 Hz, 2H). ^13^C-NMR (75 MHz, CDCl_3_, 25 °C, TMS) δ (ppm) 166.7, 146.6, 131.0, 130.6, 129.9, 128.92, 128.88, 126.6, 120.3, 55.6, 52.1, 48.1, 45.2, 28.0.

Synthesis of intermediates **50**–**51**: The corresponding methyl ester **48**–**49** (1.20 mmol) and potassium carbonate (248 mg, 1.80 mmol) were dissolved in 1:1 aqueous THF (10 mL). The solution was stirred at reflux 24 h. Hence, 1% HCl (10 mL) was added until cease of gas evolution and the solvent was removed under reduced pressure. The crude was dissolved in isopropanol (5 mL) and filtered, these steps were repeated three times. The filtrates were combined and the solvent was removed under reduced pressure.

*3-(4-(6-Carboxypyridin-2-yl)-1H-1,2,3-triazol-1-yl)-N,N-dimethylpropan-1-aminium chloride* (**50**): white solid, yield = 98%. ^1^H-NMR (300 MHz, DMSO-*d*_6_, 25 °C, TMS) δ (ppm) 8.81 (s, 1H), 8.25 (d, *J* = 7.7 Hz, 1H), 8.09 (t, *J* = 7.8 Hz, 1H), 8.00 (d, *J* = 7.6 Hz, 1H), 4.8–5.8 (bs), 4.58 (t, *J* = 6.7 Hz, 2H), 3.11 (t, *J* = 7.8 Hz, 2H), 2.78 (s, 6H), 2.33 (quint, *J* = 7.8 Hz, 2H). ^13^C-NMR (75 MHz, DMSO-*d*_6_, 25 °C, TMS) δ (ppm) 165.8, 149.9, 148.3, 146.7, 138.6, 124.2, 123.6, 122.5, 53.9, 47.0, 42.1, 24.6.

*3-(4-(3-Carboxyphenyl)-1H-1,2,3-triazol-1-yl)-N,N-dimethylpropan-1-aminium chloride* (**51**): white solid, yield = 97%. ^1^H-NMR (300 MHz, CDCl_3_, 25 °C, TMS) δ (ppm) 10.71 (bs, NH), 8.79 (s, 1H), 8.42 (s, 1H), 8.09 (d, *J* = 7.7 Hz, 1H), 7.91 (d, *J* = 7.8 Hz), 7.60 (t, *J* = 7.7 Hz, 1H), 4.55 (t, *J* = 6.7 Hz, 2H), 3.09 (t, *J* = 7.6 Hz, 2H), 2.74 (s, 6H), 2.33 (quint, *J* = 7.2 Hz, 2H). ^13^C-NMR (75 MHz, CDCl_3_, 25 °C, TMS) δ (ppm) 167.1, 145.6, 131.5, 131.1, 129.3, 128.6, 125.8, 122.1, 53.7, 47.0, 42.0, 24.5.

Fragment **33**: general method B. White solid, yield 57%. ^1^H-NMR (300 MHz, CDCl_3_, 25 °C, TMS): δ = 8.51 (s, 1H), 8.44 (bs, 1H), 8.41 (bs, 1H), 8.39 (s, 1H), 8.2 (dd, 3*J*(H, H) = 7.7 Hz, 3*J*(H, H) = 0.9 Hz, 1H), 8.01 (t, 3*J*(H, H) = 7.8 Hz, 1H), 7.80 (dd, 3*J*(H, H) = 7.7 Hz; 3*J*(H, H) = 0.9 Hz; 1H), 7.65 (t, 3*J*(H, H) = 7.8 Hz, 1H), 4.55 (t, 3*J*(H, H) = 7.0 Hz; 2H), 2.36 (t, 3*J*(H, H) = 7.0 Hz; 2H) 2.26 (s, 6H) 2.16 (quin, 3*J*(H, H) = 7.0 Hz; 2H). ^13^C-NMR (75 MHz, DMSO-*d*_6_): δ = 174.8, 167.4, 151.5, 147.0, 142.8, 138.1, 134.4, 131.4, 131.1, 129.7, 128.0, 123.3, 123.2, 123.1, 117.8, 113.3, 55.7, 48.3, 45.2, 28.0; elemental analysis calcd (%) for C_21_H_20_N_8_O: C, 62.99; H, 5.03; N, 27.98; found C, 62.92; H, 5.05; N, 28.01.

Fragment **34**: general method B. White solid, yield 61%. ^1^H-NMR (300 MHz, CDCl_3_, 25 °C, TMS): δ = 8.64 (s, 1H), 8.45 (d, 3*J*(H, H) = 8.0 Hz, 1H), 8.18 (dd, 3*J*(H, H) = 7.8 Hz, 3*J*(H, H) = 1.6 Hz, 2H), 8.08 (t, 3*J*(H, H) = 7.9 Hz, 1H), 8.01 (s, 1H), 7.87 (d, 3*J*(H, H) = 7.7 Hz, 1H), 7.63 (t, 3*J*(H, H) = 7.8 Hz, 1H), 4.54 (t, 3*J*(H, H) = 7.0 Hz; 2H), 2.37 (t, 3*J*(H, H) = 7.0 Hz; 2H) 2.28 (s, 6H) 2.16 (quint, 3*J*(H, H) = 7.0 Hz; 2H). ^13^C-NMR (75 MHz, DMSO-*d*_6_): δ = 176.6, 167.4, 148.1, 146.0, 138.4, 134.4, 132.0, 130.2, 130.0, 129.7, 127.5, 126.3, 125.3, 123.9, 120.7, 116.4, 55.6, 48.1, 45.1, 27.8; elemental analysis calcd (%) for C_21_H_20_N_8_O: C, 62.99; H, 5.03; N, 27.98; found C, 62.94; H, 5.02; N, 28.00.

Fragment **35**: general method A. White solid, yield 88%. ^1^H-NMR (300 MHz, DMSO-*d*_6_): δ = 9.9 (s, 1H), 8.93 (s, 1H), 8.57 (bs, 1H), 8.45–8.38 (m, 2H), 8.32 (dt, 3*J*(H, H) = 7.8 Hz, 3*J*(H, H) = 0.9 Hz, 1H), 8.22 (dd, 3*J*(H, H) = 7.7 Hz, 3*J*(H, H) = 1 Hz, 1H), 8.02 (dd, 3*J*(H, H) = 7.7 Hz, 3*J*(H, H) = 1 Hz, 1H), 7.73 (t, 3*J*(H, H) = 7.8 Hz, 1H), 6.1 (s, 2H), 4.59 (t, 3*J*(H, H) = 7.0 Hz; 2H), 2.34 (t, 3*J*(H, H) = 7.0 Hz; 2H) 2.26 (s, 6H) 2.14 (quin, 3*J*(H, H) = 7.0 Hz; 2H). ^13^C-NMR (75 MHz, DMSO-*d*_6_): δ = 174.3, 168.3, 151.0, 150.2, 146.0, 142.7, 139.3, 134.4, 129.3, 128.5, 127.4, 125.9, 124.3, 123.5, 123.0, 55.7, 47.9, 45.1, 27.7; elemental analysis calcd (%) for C_21_H_23_N_9_O_2_: C, 58.19; H, 5.35; N, 29.08; found C, 58.24; H, 5.38; N, 29.05.

Fragment **36**: general method A. White solid, yield 82%. ^1^H-NMR (300 MHz, DMSO-*d*_6_): δ = 10.16 (s, 1H), 8.85 (s, 1H), 8.68 (bs, 1H), 8.31–8.19 (m, 3H), 8.09 (d, 3*J*(H, H) = 4.4 Hz, 2H), 7.77 (t, 3*J*(H, H) = 7.8 Hz, 1H), 5.92 (s, 2H) 4.47 (t, 3*J*(H, H) = 7.0 Hz; 2H), 2.32 (t, 3*J*(H, H) = 7.0 Hz; 2H) 2.20 (s, 6H) 2.06 (quin, 3*J*(H, H) = 7.0 Hz; 2H). ^13^C-NMR (75 MHz, DMSO-*d*_6_): δ = 176.0, 168.4, 151.0, 149.2, 145.3, 144.8, 138.6, 132.5, 130.7, 130.1, 127.5, 124.5, 124.4, 124.0, 122.7, 122.1, 55.8, 48.1, 45.2, 27.7; elemental analysis calcd (%) for C_21_H_23_N_9_O_2_: C, 58.19; H, 5.35; N, 29.08; found C, 58.23; H, 5.36; N, 29.02.

### 4.3. Biophysical and Biological Assays

#### 4.3.1. FRET Assay

For fluorescence melting curves, 6-carboxyfluorescein (FAM) 5′-end and 6-carboxy-tetramethylrhodamine (TAMRA) 3′-end labelled oligonucleotides (0.25 μM) (Tabel S1) were folded in lithium cacodylate buffer (10 mM, pH 7.4) and KCl 100 mM by heating at 95 °C for 5 min and gradually cooling to r.t.. Where indicated, fragments were added at the final concentration of 25 μM or 1 mM and, after stabilization at 4 °C, samples were processed in a LightCycler 480 II (Roche, Milan, Italy). Oligonucleotide melting was monitored by observing FAM emission in the temperature range of 30–95 °C with 1 °C/min gradient. Melting profiles were normalized as previously described [47] and T_m_ was defined as the temperature corresponding to the 0.5 fraction of the normalized fluorescence.

For competition assays, 5′-FAM and 3′-TAMRA labelled LTR-III oligonucleotide and unlabelled oligonucleotides (competitors) (Tabel S1) were separately folded for 5 min at 95 °C in lithium cacodylate buffer (10 mM, pH 7.4) supplemented with KCl (100 mM). After 4 h at r.t., labelled oligonucleotide (0.25 µM) was mixed with increasing amounts of competitor (0–32-fold excess) in the presence of **36** (25 µM). Samples were processed by Light Cycler (Roche, Milan, Italy) and T_m_ were obtained as described above.

#### 4.3.2. Circular Dichroism (CD)

For CD analysis, oligonucleotides (Table S1) were diluted to a final concentration of 1.5 µM in lithium cacodylate buffer (10 mM, pH 7.4) and KCl 100 mM. Samples were annealed by heating at 95 °C for 5 min and gradually cooled to r.t. and, where indicated, fragments were added at the final concentration of 15 μM. CD spectra were recorded on a Chirascan-Plus (Applied Photophysics, Leatherhead, UK) equipped with a Peltier temperature controller using a quartz cell of 5 mm optical path length, over a wavelength range of 230–320 nm. The reported spectrum of each sample represents the average of 2 scans at 20 °C and it is baseline corrected for signal contributions due to the buffer. Observed ellipticities were converted to mean residue ellipticity (θ) = deg × cm^2^ × dmol^−1^ (mol. ellip.). For the determination of T_m_, spectra were recorded over a temperature range of 20–90 °C, with temperature increase of 5 °C. T_m_ values were calculated according to the van’t Hoff equation, applied for a two-state transition from folded to unfolded state, assuming that the heat capacity of the folded and unfolded states are equal [48]. 

#### 4.3.3. Taq Polymerase Stop Assay

The DNA primer (Table S1) was 5′-end labelled with [γ-^32^P]ATP using T4 polynucleotide kinase (Thermo Scientific, Milan, Italy) at 37 °C for 30 min and then purified with Illustra MicroSpin G-25 columns (GE Healthcare, Milan, Italy). The labelled primer (final concentration 72 nM) was annealed to the template (final concentration 36 nM) (Table S1) in lithium cacodylate buffer (10 mM, pH 7.4) in the presence or absence of KCl 100 mM by heating at 95 °C for 5 min and gradually cooling to r.t. to allow both primer annealing and G4 folding. Where specified, **35**, **36** or NDI were added at the indicated concentrations and incubated overnight. Primer extension was performed with 2 U/reaction of AmpliTaq Gold DNA polymerase (Applied Biosystem, Carlsbad, CA, USA) at 42 °C for 30 min. Reactions were stopped by ethanol precipitation and primer extension products were separated on a 16% denaturing gel and finally visualized by phosphorimaging (Typhoon FLA 9000, GE Healthcare, Milan, Italy). Markers were prepared based on Maxam & Gilbert sequencing by PCR reaction with ^32^P-labeled primer. PCR products were treated with formic acids for 5 min at 25 °C and then with piperidin for 30 min at 90 °C.

## Figures and Tables

**Table 1:** Stabilization of LTR-III, hTel G4s and dsDNA (0.25 μM) in the presence of 4000-fold excess of fragments (1 mM) vs. oligonucleotide, measured by FRET melting assay.

Fragment	Structural Complexity	ΔT_m_^1^ ± s.d. ^2^ (°C)		
		LTR-III	hTel	dsDNA
**1**	a	<1	<1	<1
**2**		<1	<1	<1
**3**		<1	<1	<1
**4**		<1	<1	<1
**5**		1.5 ± 0.1	1.0 ± 0.1	<1
**6**		<1	<1	<1
**7**		<1	<1	<1
**8**		<1	<1	<1
**9**		<1	<1	<1
**10**		<1	<1	<1
**11**		1.5 ± 0.1	1.5 ± 0.1	<1
**12**		1.5 ± 0.1	1.5 ± 0.1	<1
**13**		<1	<1	<1
**14**		6.0 ± 1.5	4.5 ± 2.2	2.5 ± 0.5
**15**		1.5 ± 0.1	3.0 ± 0.5	<1
**16**	b	3.6 ± 0.6	2.5 ± 0.1	<1
**17**		1.5 ± 0.1	3.0 ± 0.5	<1
**18**		<1	<1	<1
**19**		n.d. ^3^	n.d. ^3^	n.d. ^3^
**20**		5.5 ± 0.5	5.5 ± 0.5	<1
**21**		n.d. ^3^	n.d. ^3^	n.d. ^3^
**22**		5.5 ± 0.5	4.0 ± 0.5	<1
**23**		4.1 ± 1.3	2.7 ± 1.1	<1
**24**		7.1 ± 0.1	6.5 ± 0.8	1.1 ± 0.2
**25**		4.5 ± 0.5	2.5 ± 0.1	<1
**26**		6.5 ± 0.5	5.0 ± 0.5	<1
**27**		13.0 ± 0.6	14.0 ±0.7	<1
**28**		6.0 ± 0.5	4.5 ± 0.5	<1
**29**		3.0 ± 0.6	5.4 ± 0.9	1.1 ± 0.2
**30**		10.8 ± 1.0	14.7 ± 1.1	<1
**31**		n.d. ^3^	n.d. ^3^	n.d. ^3^
**32**		12.8 ± 2.0	11.8 ± 1.9	<1
**33**	c	>22.1	>24.1	>28.0
**34**		>22.1	>24.1	>28.0
**35**		>22.1	>24.1	4.1 ± 0.2
**36**		>22.1	>24.1	14.1 ± 1.0

^1^ Variation in the oligonucleotide T_m_ in the presence of the selected compound. ^2^ Standard deviation. ^3^ Not determinable.

**Table 2:** Stabilization of LTR-III, hTel G4s and dsDNA (0.25 μM) in the presence of 100-fold excess of fragments (25 μM) vs. oligonucleotide, measured by FRET melting assay.

Fragment	Structural Complexity	ΔT_m_^1^ ± s.d. ^2^ (°C)		
		LTR-III	hTel	dsDNA
(**16**)	b	<1	<1	<1
(**23**)		<1	<1	<1
(**24**)		2.9 ± 0.3	1.1 ± 0.1	<1
(**29**)		<1	<1	<1
(**30**)		3.5 ± 0.5	3.0 ± 0.5	<1
(**32**)		5.3 ± 0.5	3.5 ± 0.4	<1
(**33**)	c	15.5 ± 0.5	15.3 ± 0.3	<1
(**34**)		14.3 ± 0.3	14.0 ± 1.0	<1
(**35**)		9.0 ± 0.2	8.9 ± 0.1	<1
(**36**)		14.1 ± 0.2	14.6 ± 0.3	<1

^1^ Variation in the oligonucleotide T_m_ in the presence of the selected compound. ^2^ Standard deviation.

## Supplementary Materials

The following are available online. Table S1: Oligonucleotides used in this study, NMR Figures: ^1^H and ^13^C-NMR characterization of all compounds presented in the main text.
