# Supplementary material for: A Fragment-Based Approach for the Development of G-Quadruplex Ligands: Role of the Amidoxime Moiety
Source: Molecules. 2018 Jul 27;23(8):1874. doi: 10.3390/molecules23081874 (PMC6222391; doi:10.3390/molecules23081874)

A fragment-based approach for the development of G-quadruplex ligands: role of the amidoxime moiety

Martina Tassinari[1], Alberto Lena[2], Elena Butovskaya[1], Valentina Pirota[2], Matteo Nadai[1], Mauro Freccero[2], Filippo Doria[2,*] and Sara N. Richter[1,*]

1 Department of Molecular Medicine, University of Padua, via A. Gabelli 63, 35121 Padua, Italy; martina.tassinari@unipd.it ; elena.butovskaya@gmail.com ; matteo.nadai@unipd.it ; sara.richter@unipd.it

2 Department of Chemistry, University of Pavia, Viale Taramelli 10, 27100 Pavia, Italy; alberto.lena01@ateneopv.it ; valentina.pirota01@universitadipavia.it ; mauro.freccero@unipv.it ; filippo.doria@unipv.it

* Correspondence: sara.richter@unipd.it; Tel.: +39-049-8272446; filippo.doria@unipv.it

Supplementary Material

Table of Contents:

Table S1. Oligonucleotides used in this study page S2

NMR Figures. ^{1}H and ^{13}C NMR characterization of all compounds presented in the main text page S3-S23

Table S1. Oligonucleotides used in this study

Application	Name	Sequence (5′→ 3′)
FRET melting	LTR-III	FAM[1]-GGGAGGCGTGGCCTGGGCGGGACTGGGG-TAMRA[2]
	hTel	FAM[1]-AGGGTTAGGGTTAGGGTTAGGG-TAMRA[2]
	dsDNA	FAM[1]-CTATAGCGCGCTATAG-TAMRA[2]
FRET competition	LTR-III labeled	FAM[1]-GGGAGGCGTGGCCTGGGCGGGACTGGGG-TAMRA[2]
	LTR-III unlabeled	GGGAGGCGTGGCCTGGGCGGGACTGGGG
	hTel unlabeled	AGGGTTAGGGTTAGGGTTAGGG
CD	LTR-III	GGGAGGCGTGGCCTGGGCGGGACTGGGG
	hTel	AGGGTTAGGGTTAGGGTTAGGG
Taq polymerase stop assay	Taq primer	GGCAAAAAGCAGCTGCTTATATGCAG
	non-G4 cnt	TTGTCGTTAAAGTCTGACTGCGAGCTCTCAGATCCTGCATAT<br>AAGCAGCTGCTTTTTGCC
	LTR-III	TTTTTGGGAGGCGTGGCCTGGGCGGGACTGGGGTTTTTCTGC<br>ATATAAGCAGCTGCTTTTTGCC
	hTel	TTTTTGGGTTAGGGTTAGGGTTAGGGTTTTTCTGCATATAAGC<br>GCTTTTTGCC

[1] 6-carboxyfluorescein. [2] 6-carboxy-tetramethylrhodamine.

NMR characterization:

^{1}H-NMR – DMSO d6: Fragment **5**

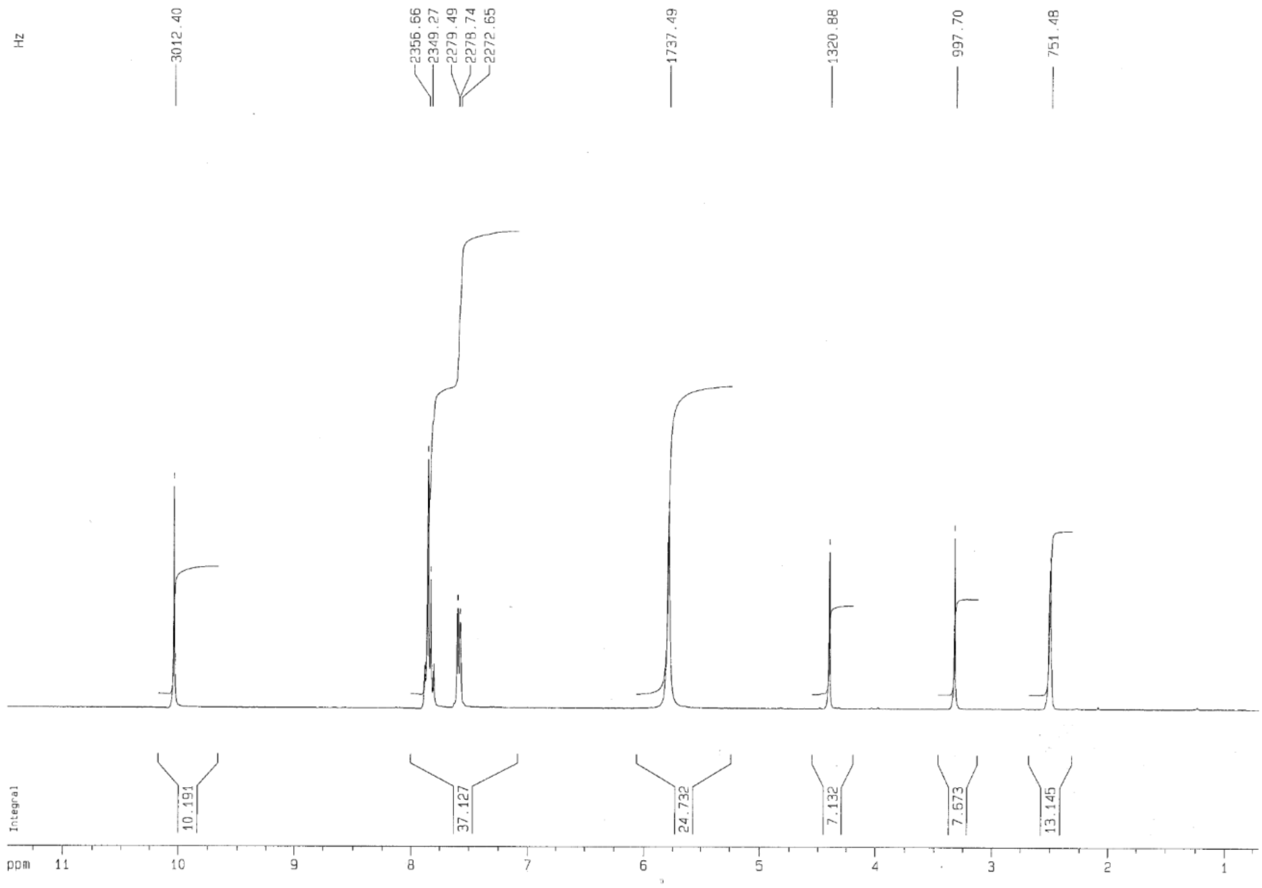

^{13}C-NMR – DMSO d6: Fragment **5**

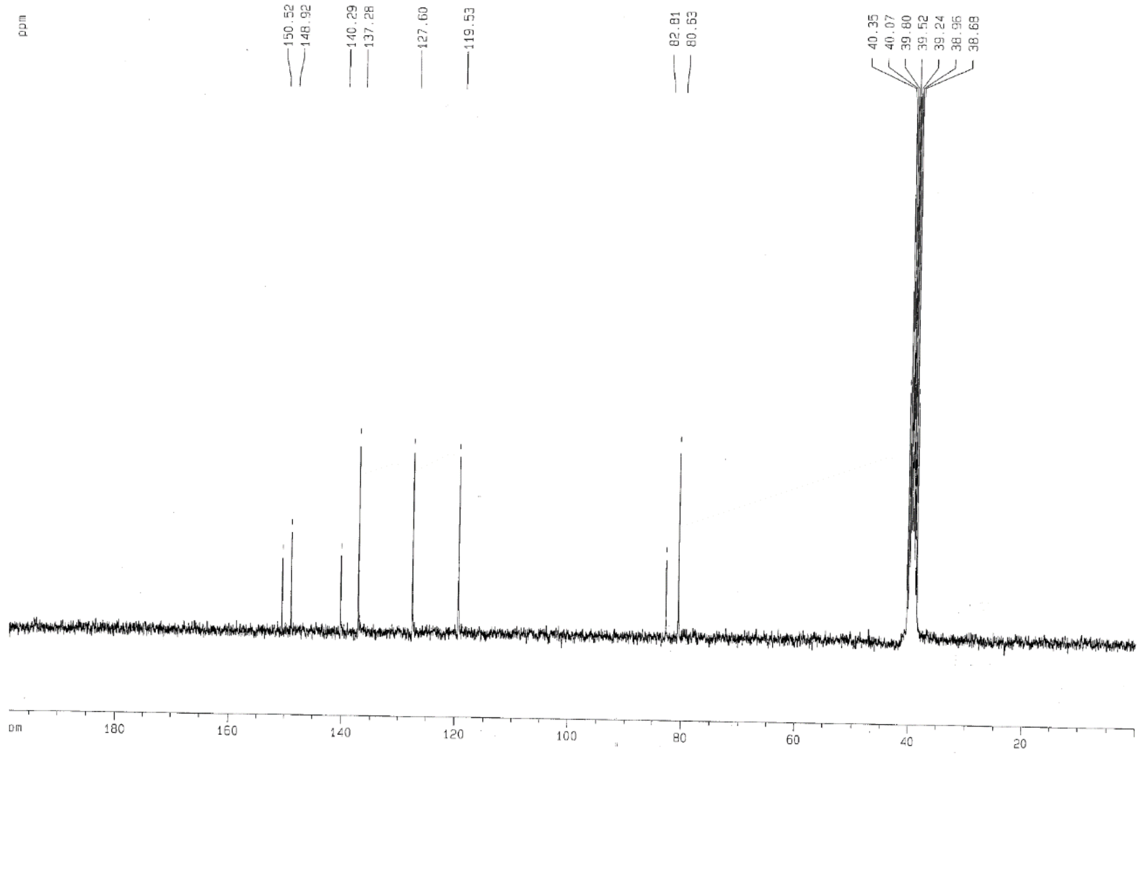

^{1}H-NMR – DMSO d6: Fragment **16**

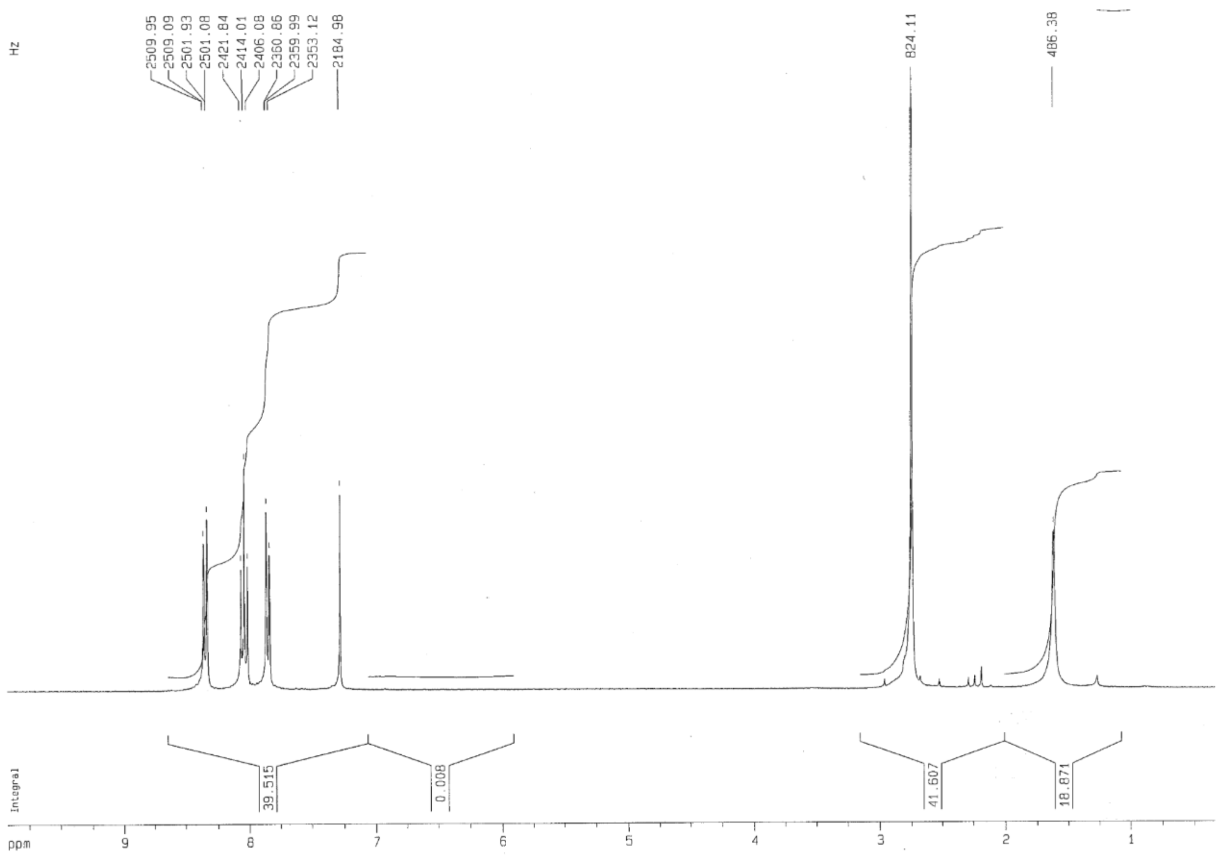

^{13}C-NMR – DMSO d6: Fragment **16**

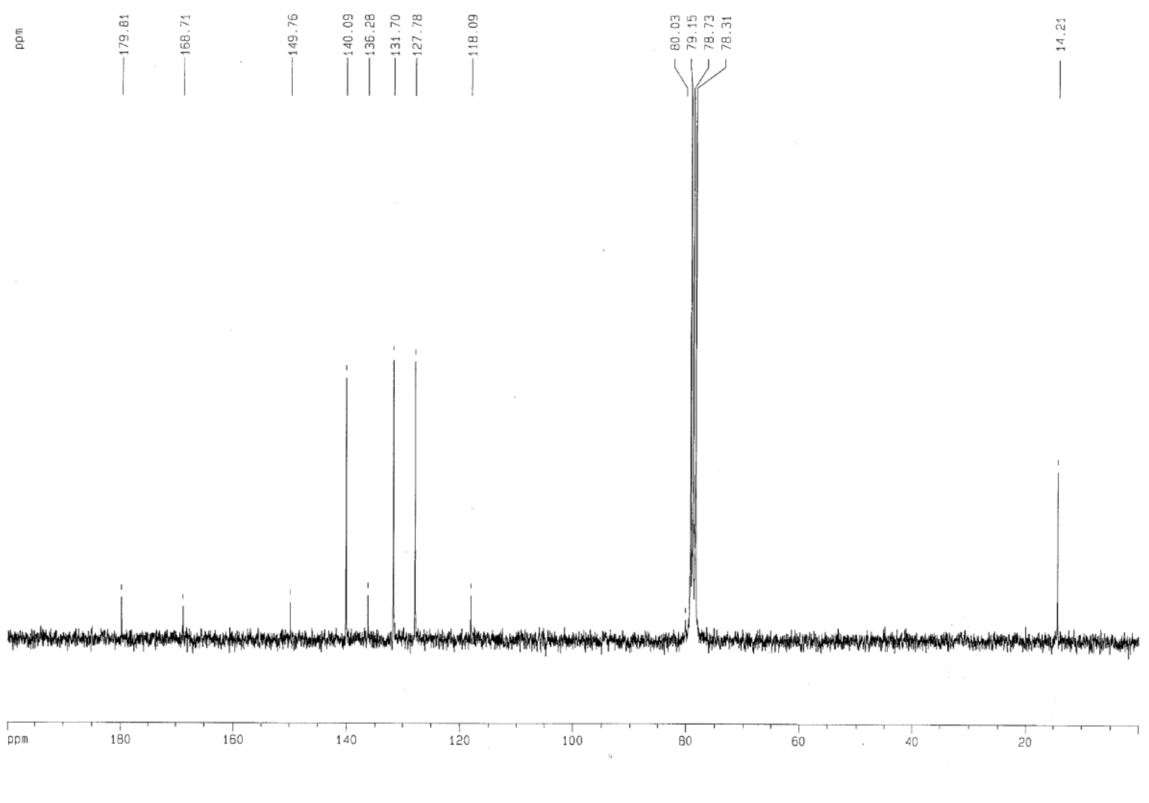

^{1}H-NMR – $CDCl_3$: Fragment **17**

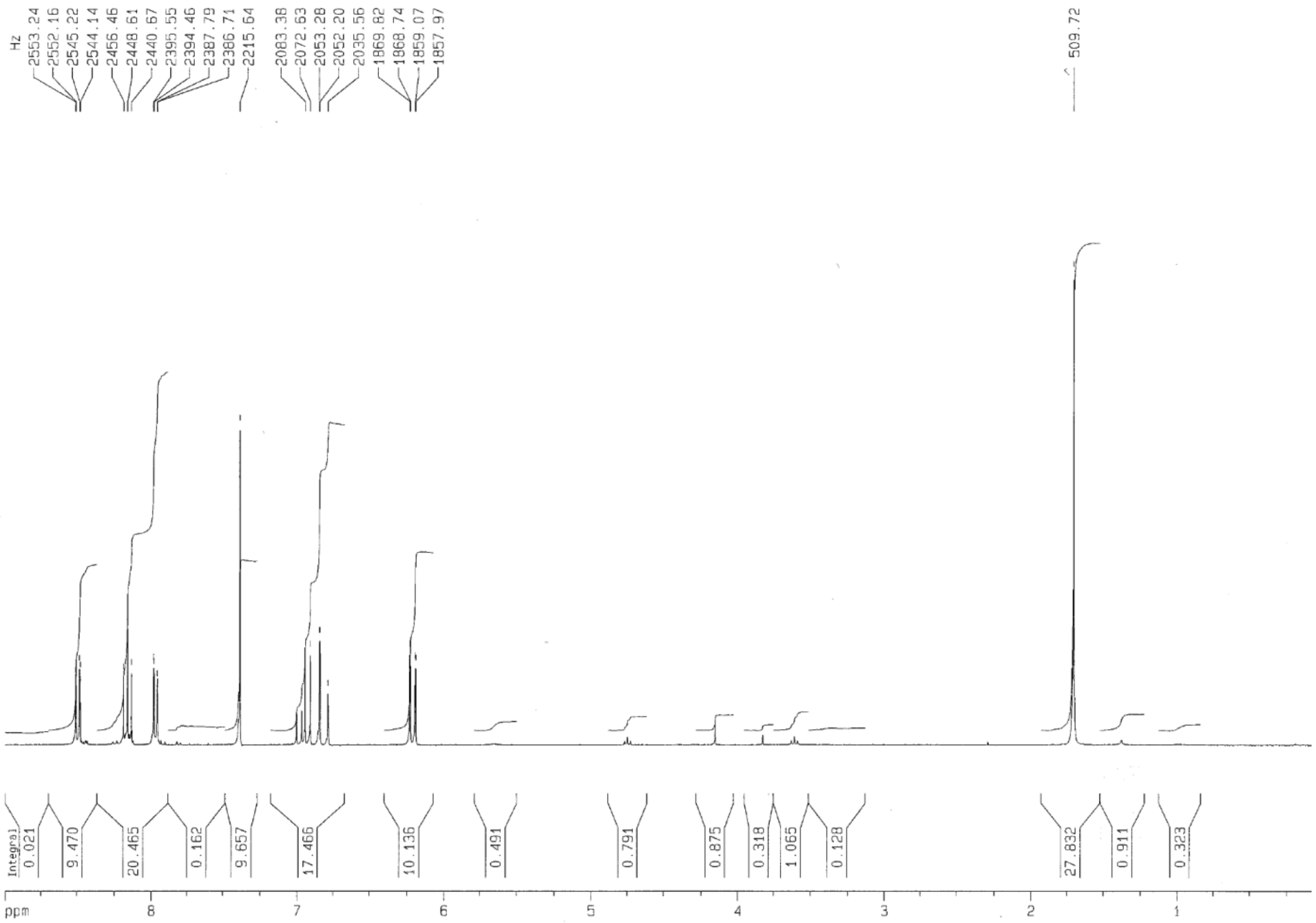

^{13}C-NMR – $CDCl_3$: Fragment **17**

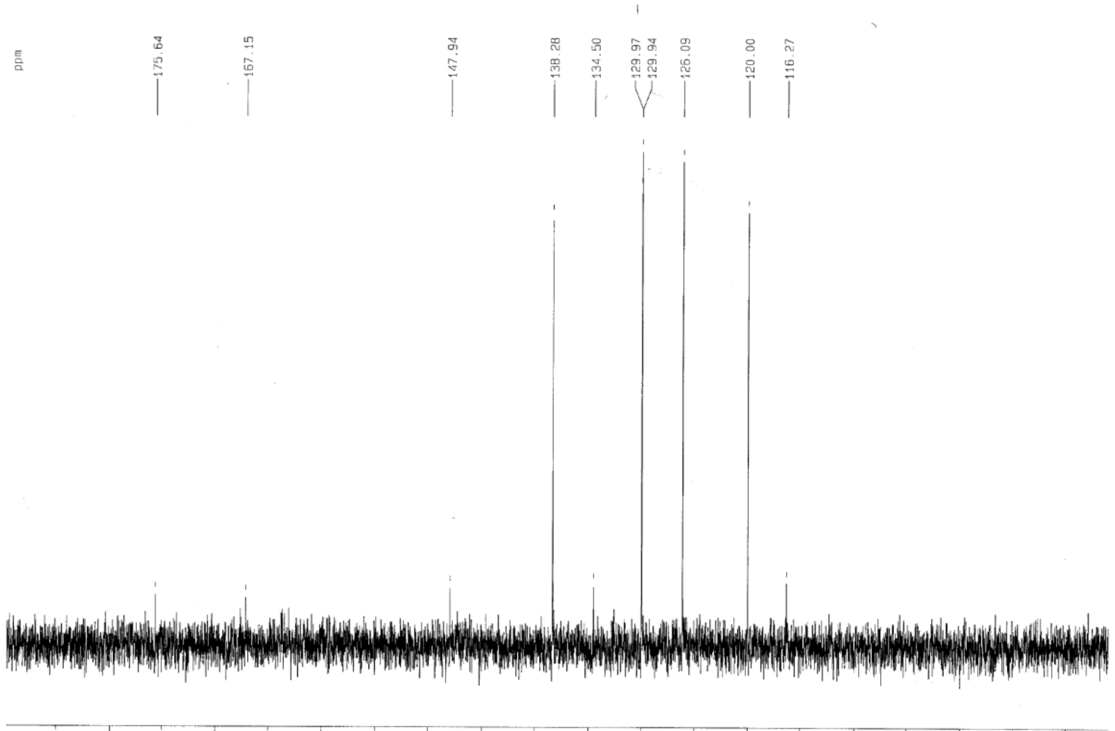

^{1}H-NMR – DMSO d6: Fragment **18**

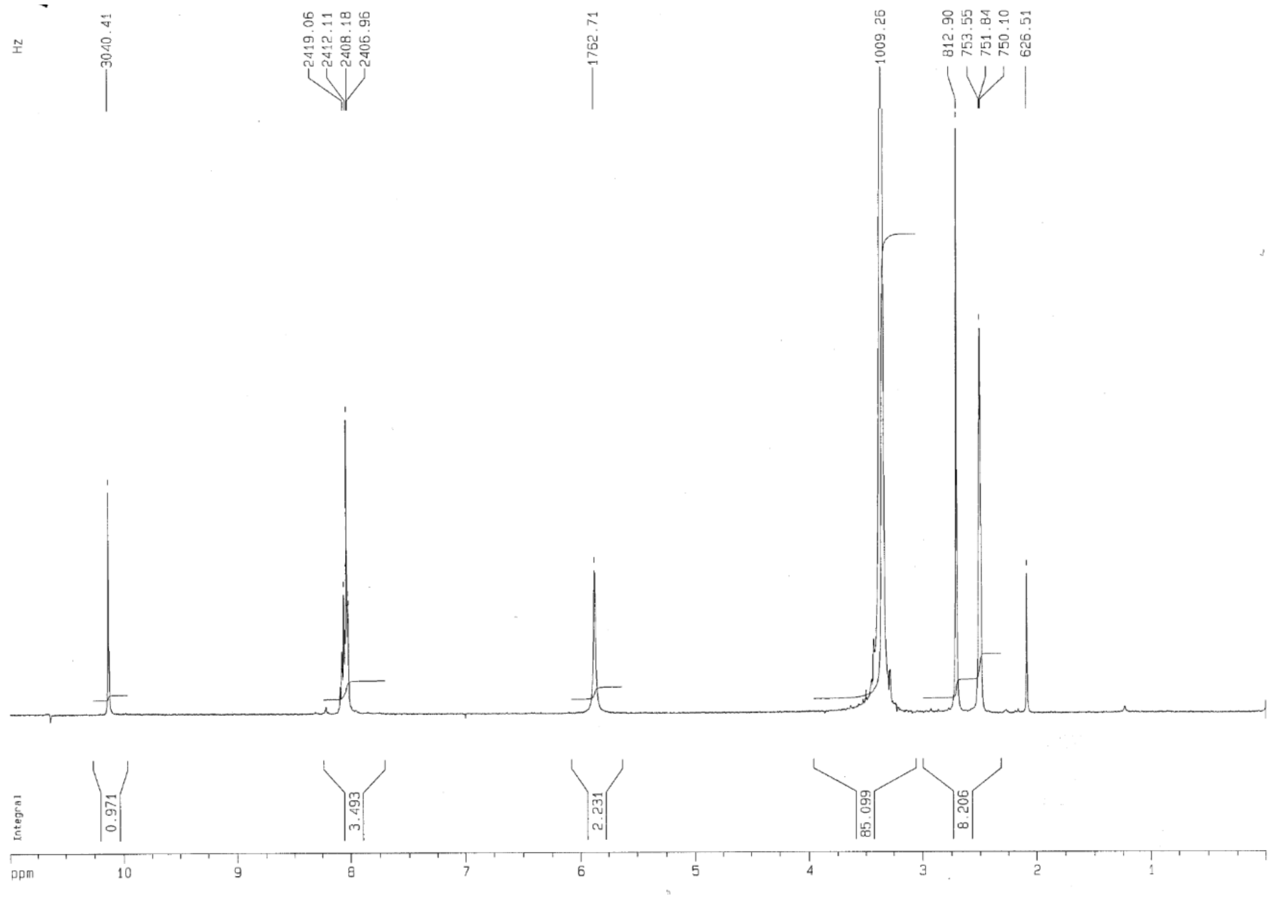

^{13}C-NMR – DMSO d6: Fragment **18**

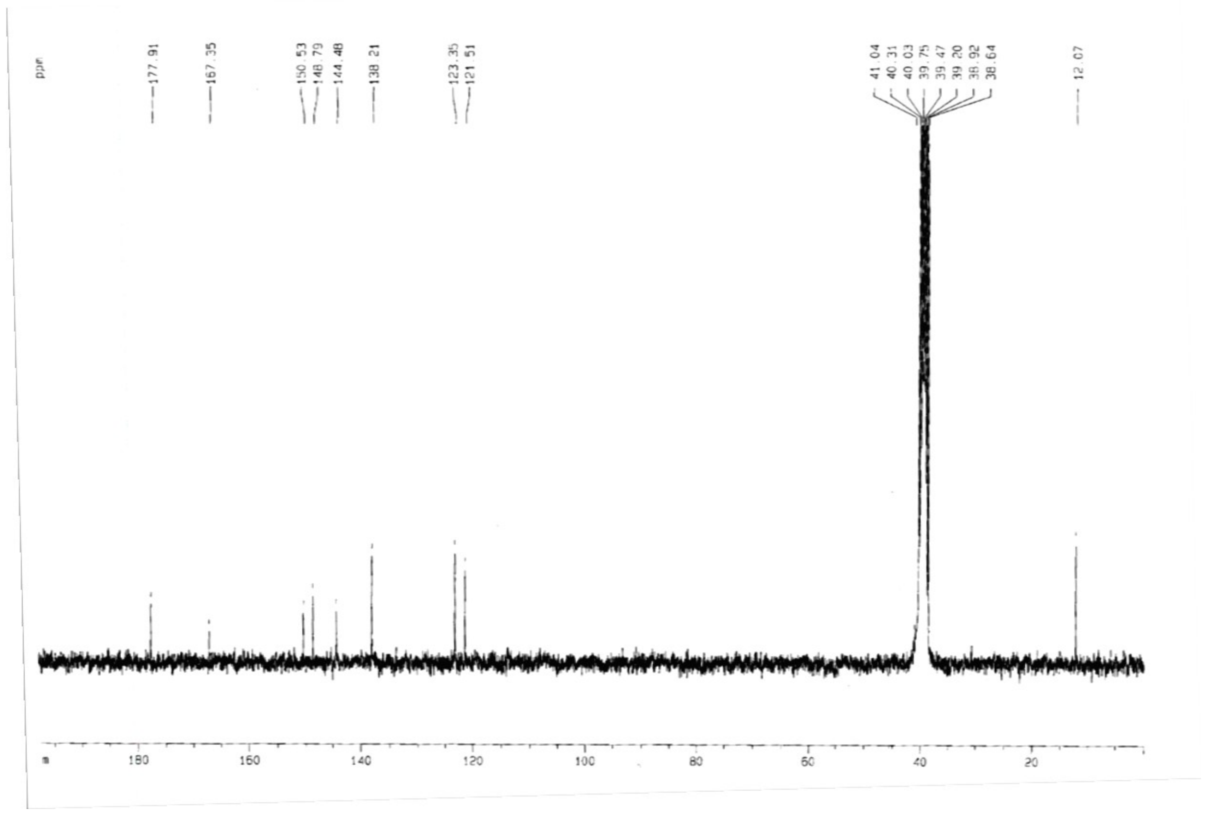

^{1}H-NMR – DMSO d6: Fragment **19**

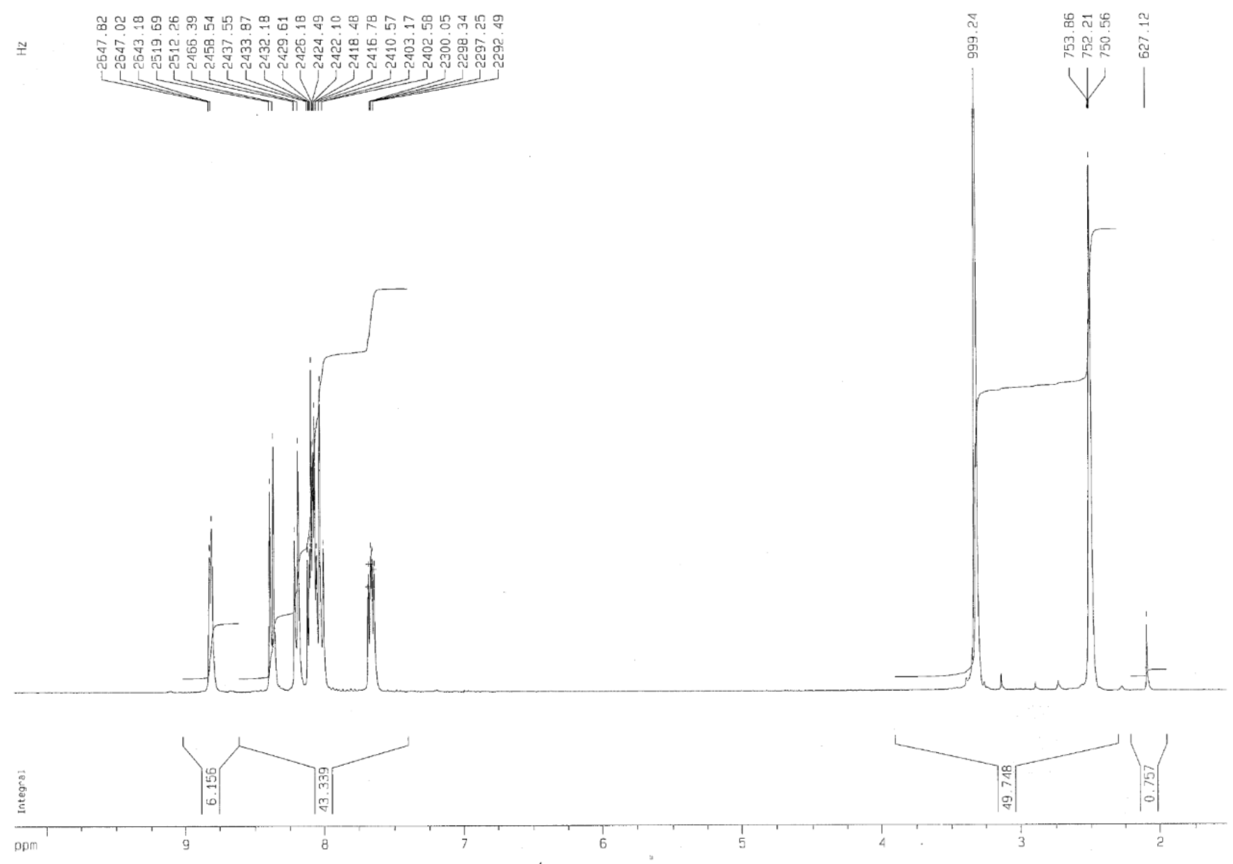

^{13}C-NMR – DMSO d6: Fragment **19**

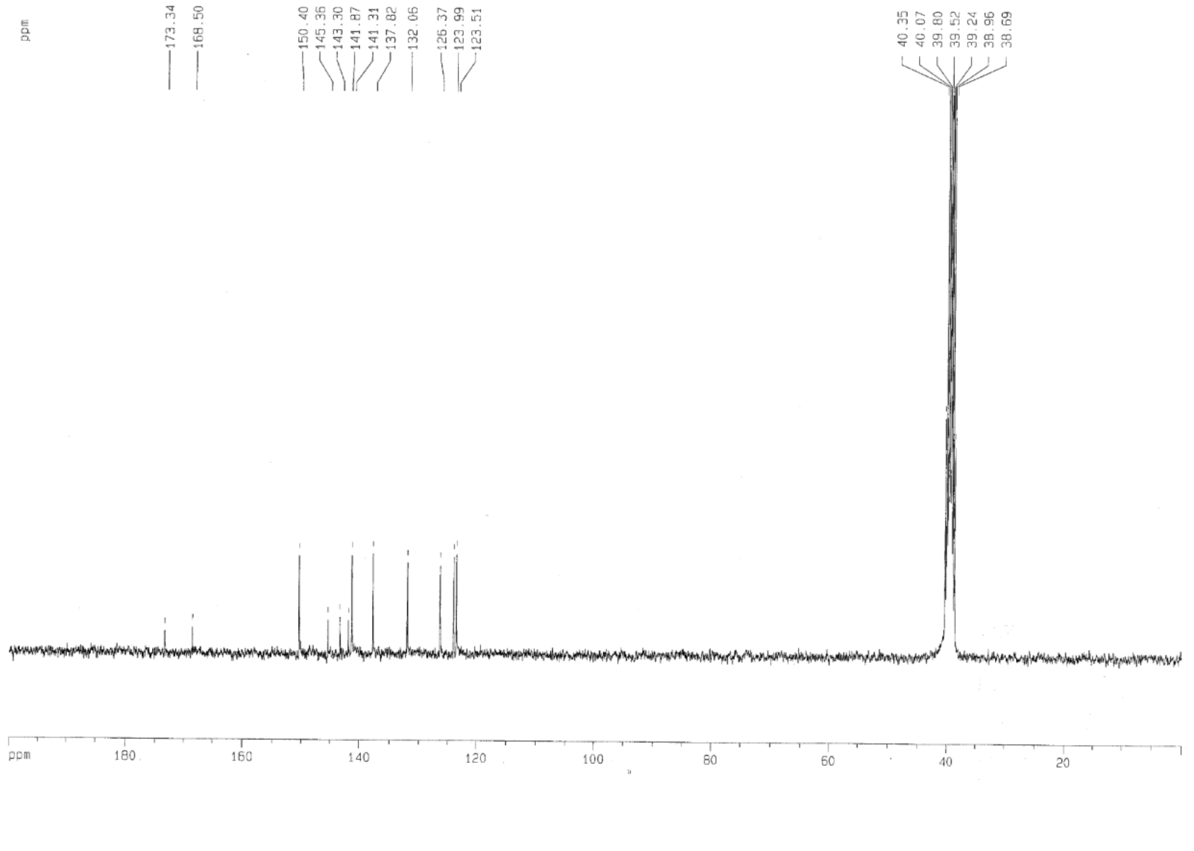

^{1}H-NMR – DMSO d6: Fragment **20**

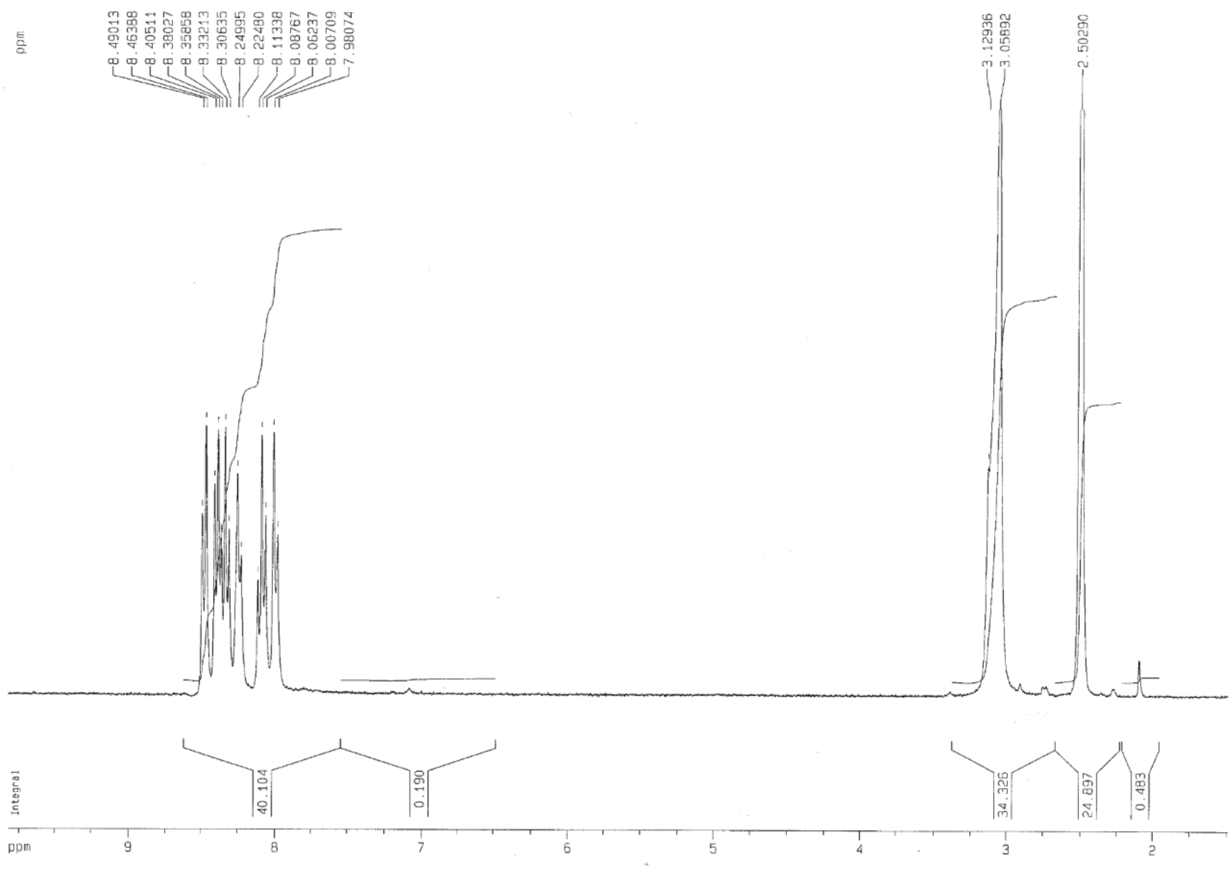

^{13}C-NMR – DMSO d6: Fragment **20**

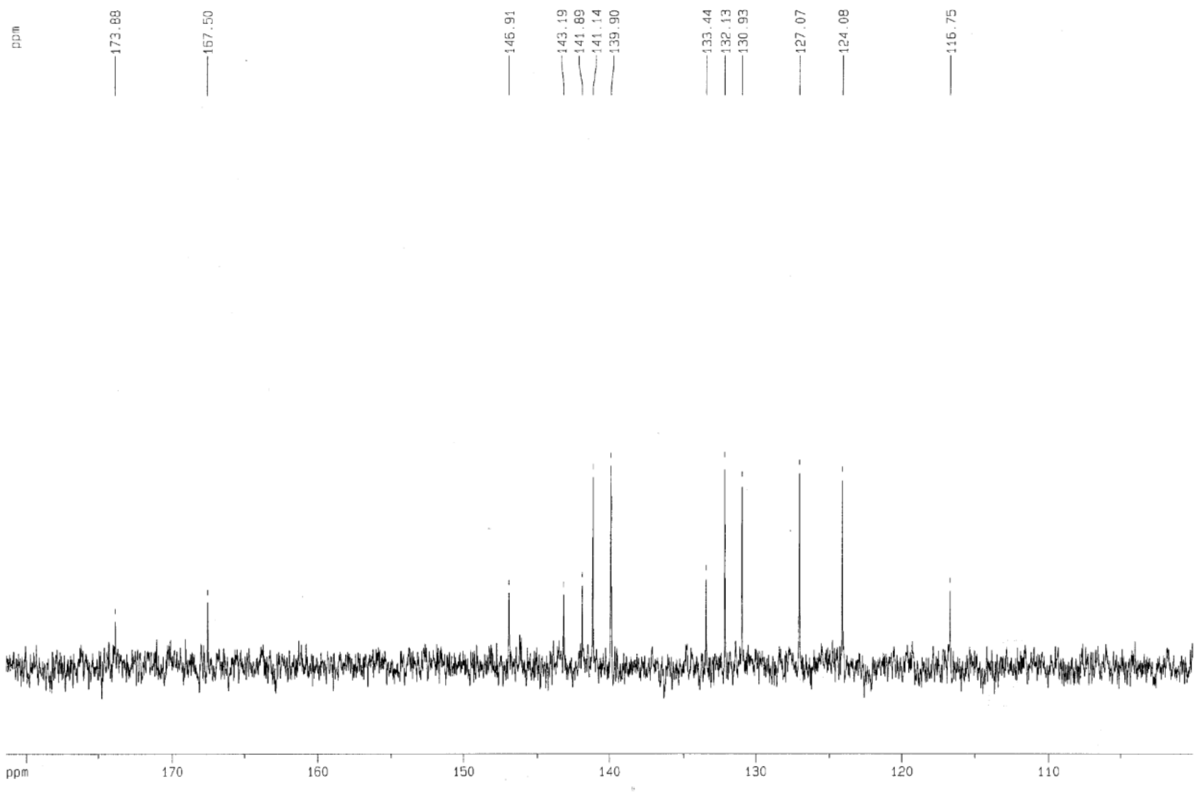

^{1}H-NMR – $CDCl_3$: Fragment **21**

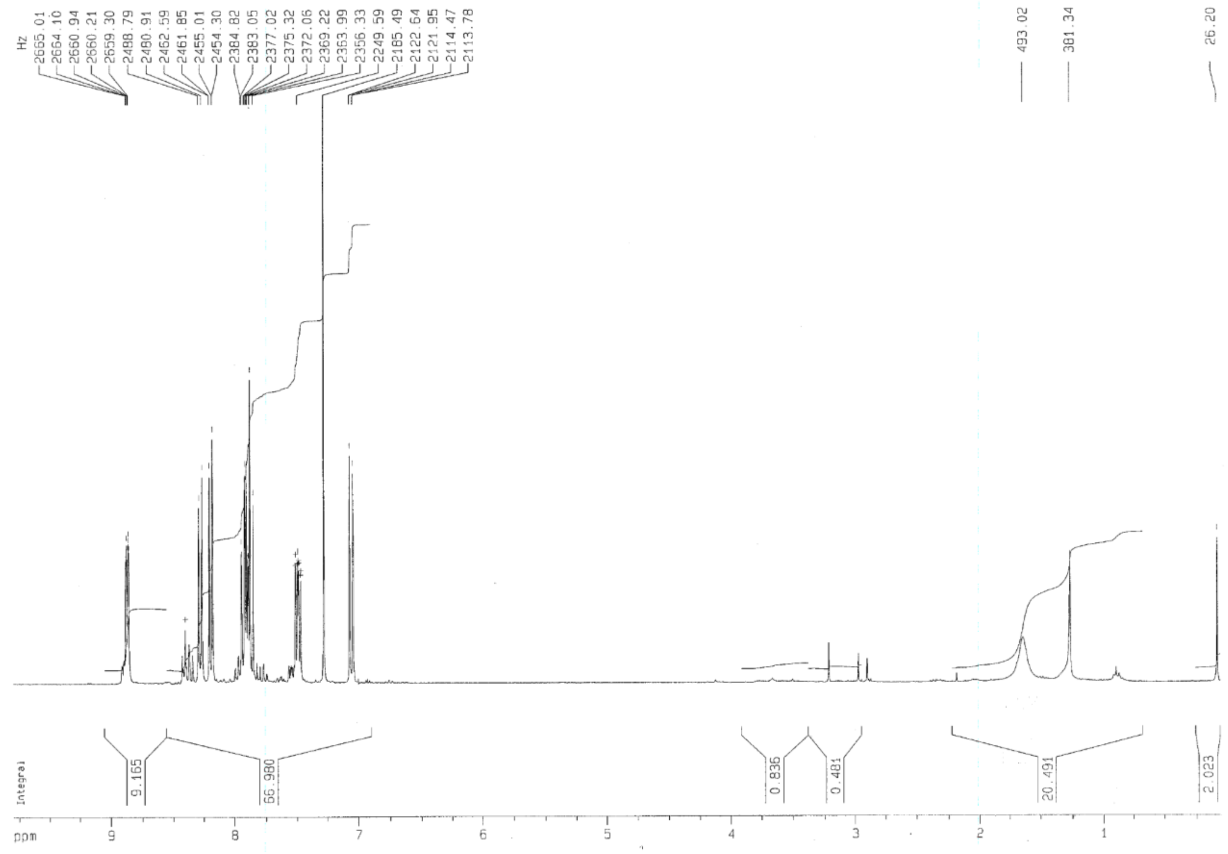

^{13}C-NMR – $CDCl_3$: Fragment **21**

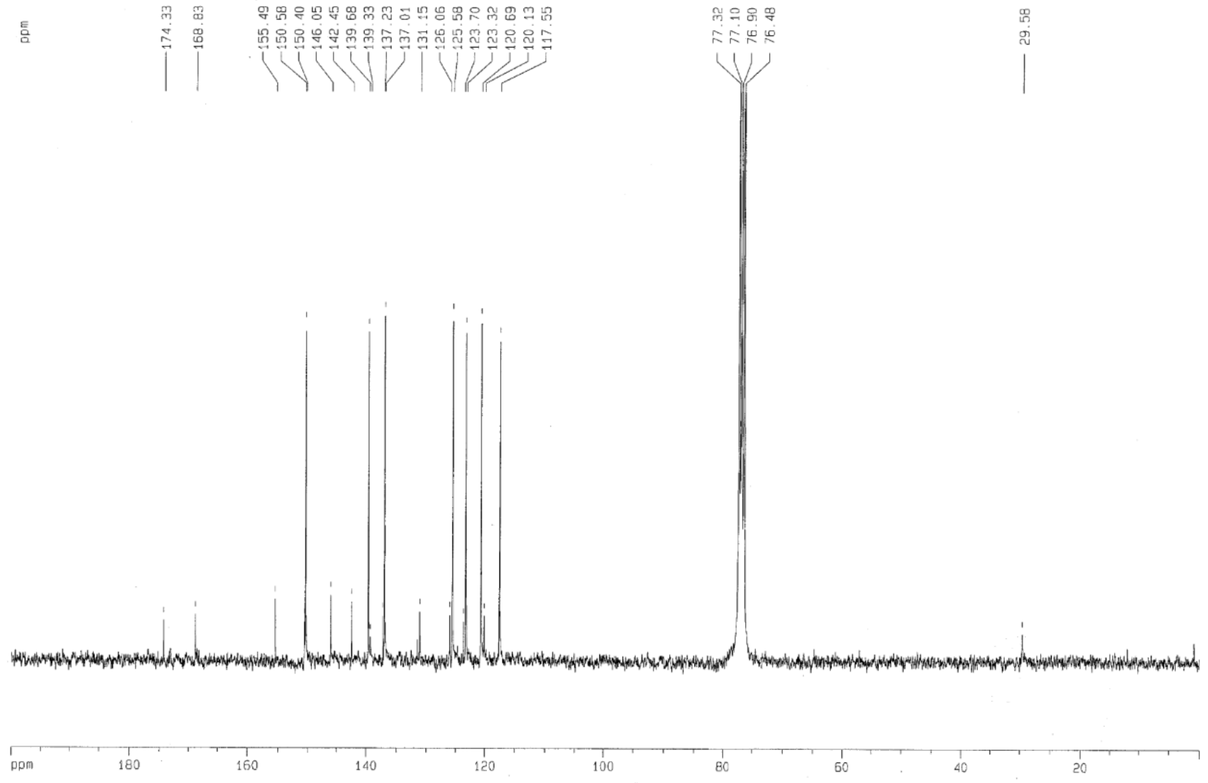

^{1}H-NMR – DMSO d6: Fragment **22**

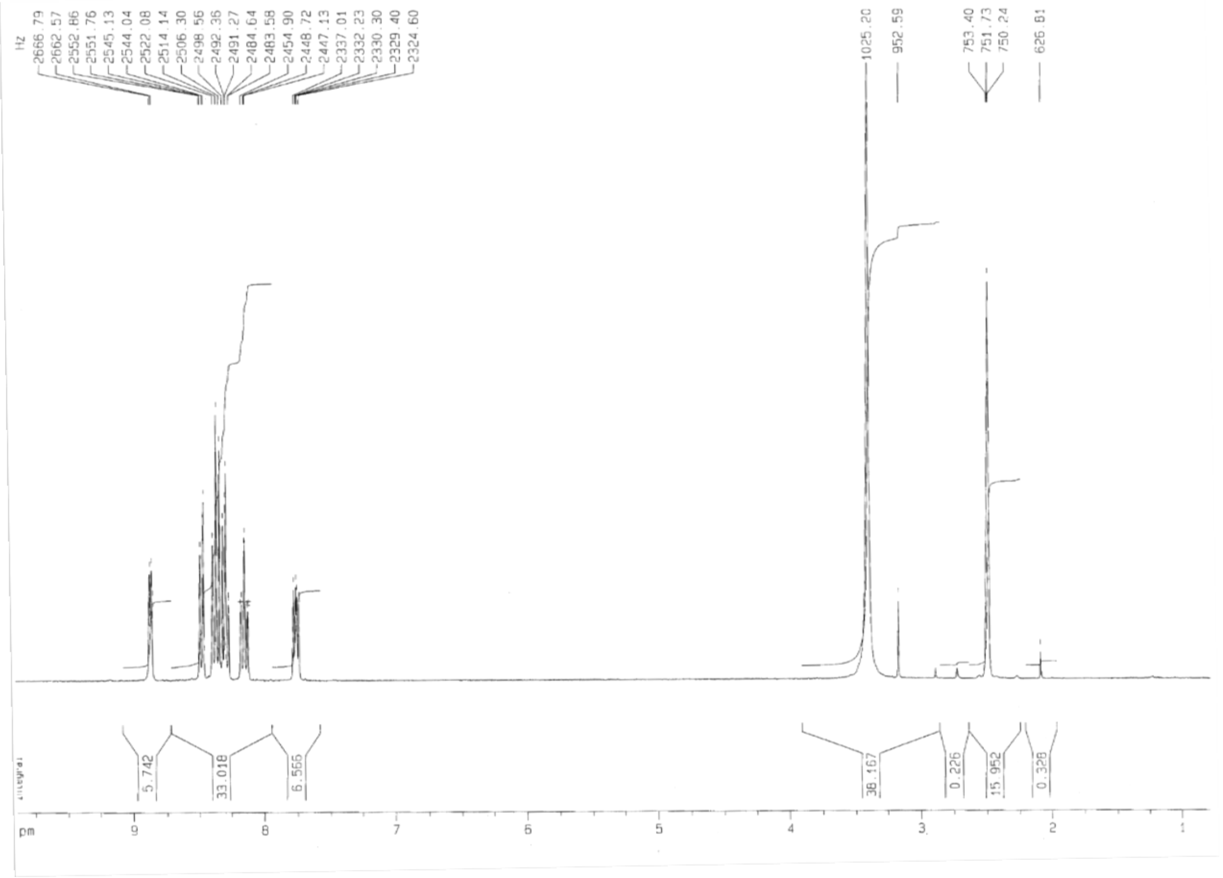

^{13}C-NMR – DMSO d6: Fragment **22**

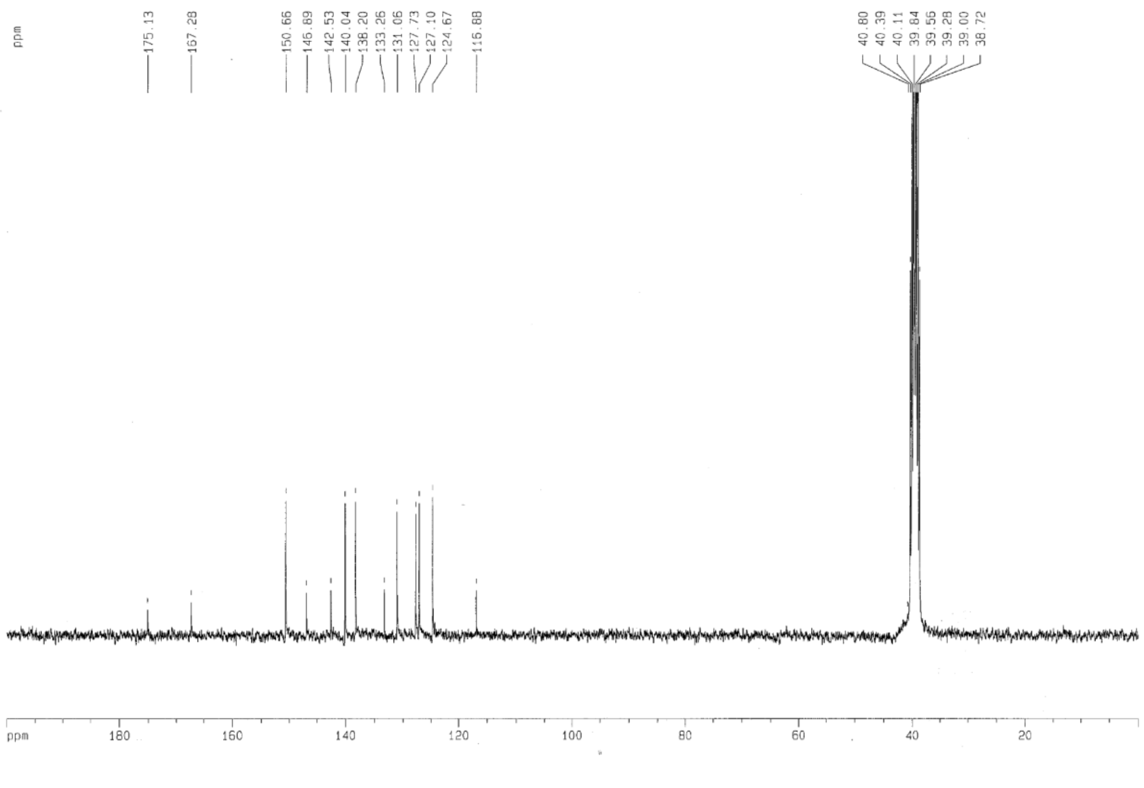

^{1}H-NMR – DMSO d6: Fragment **23**

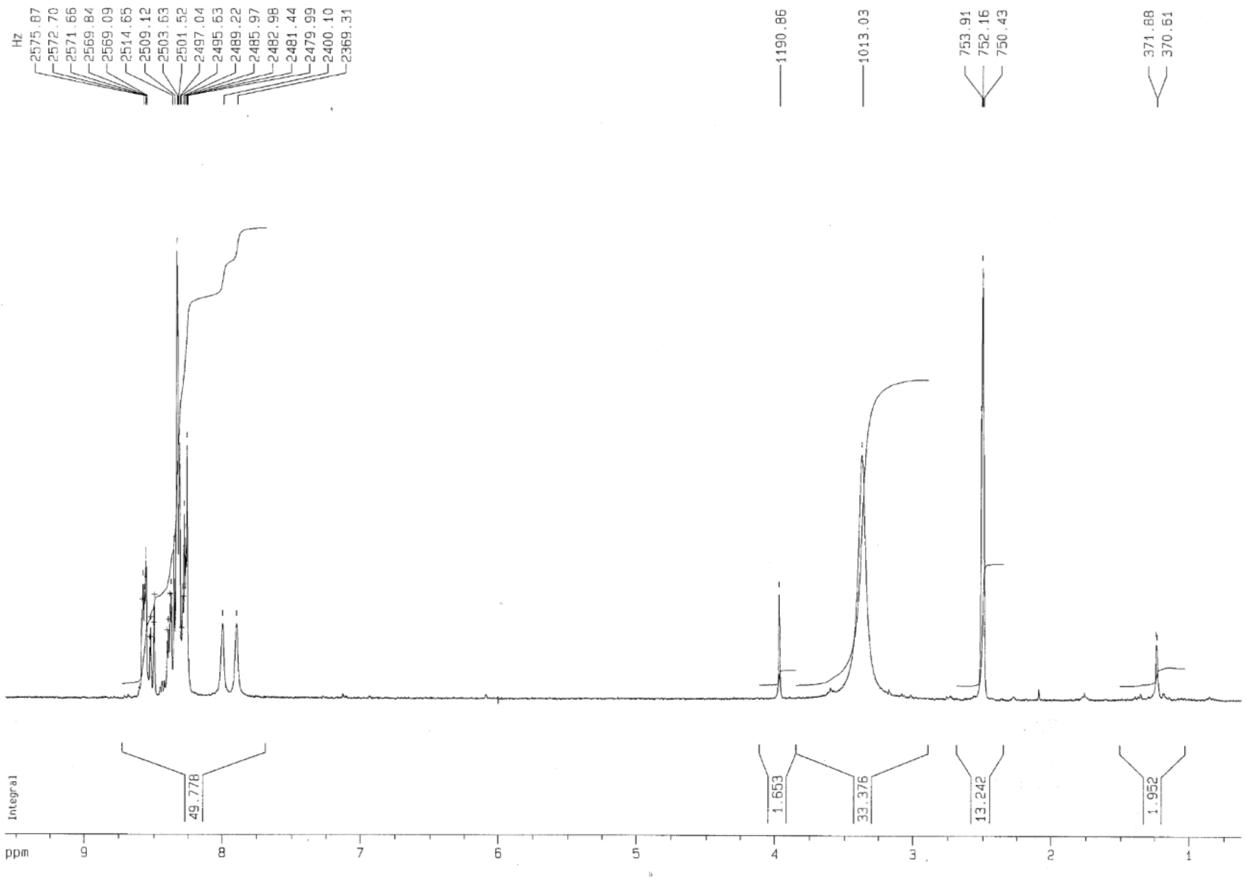

^{13}C-NMR – DMSO d6: Fragment **23**

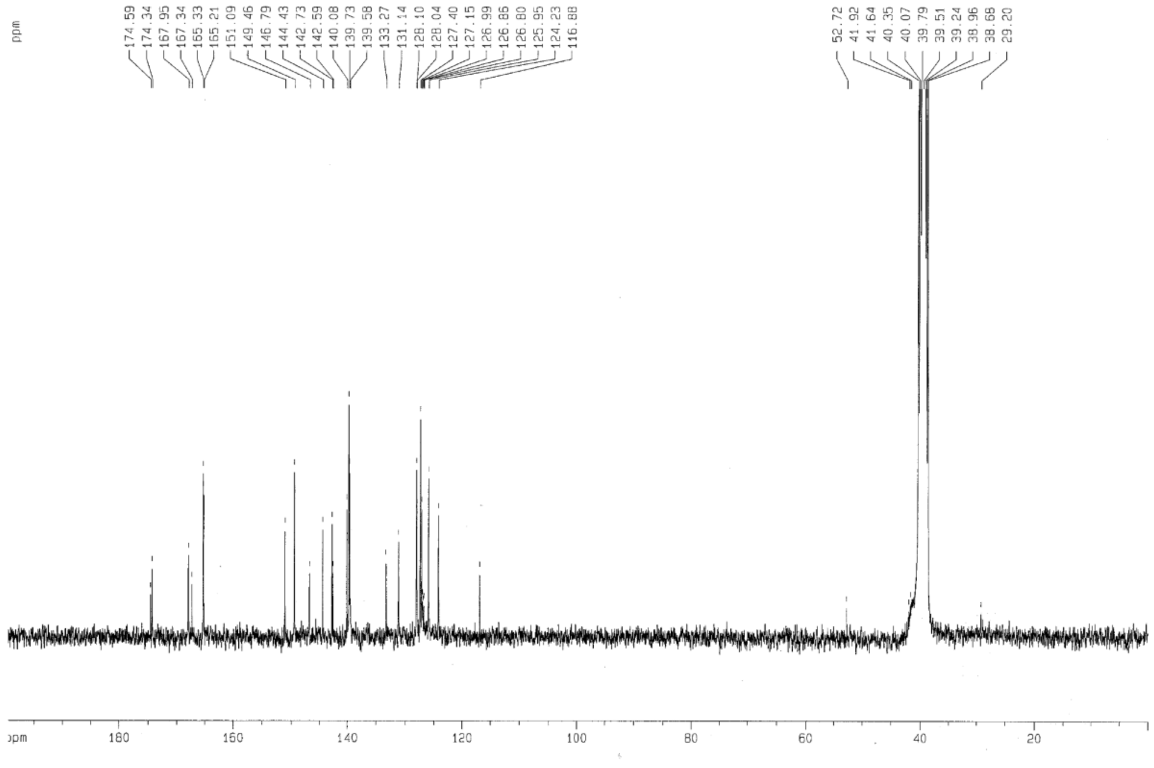

^{1}H-NMR – DMSO d6: Fragment **24**

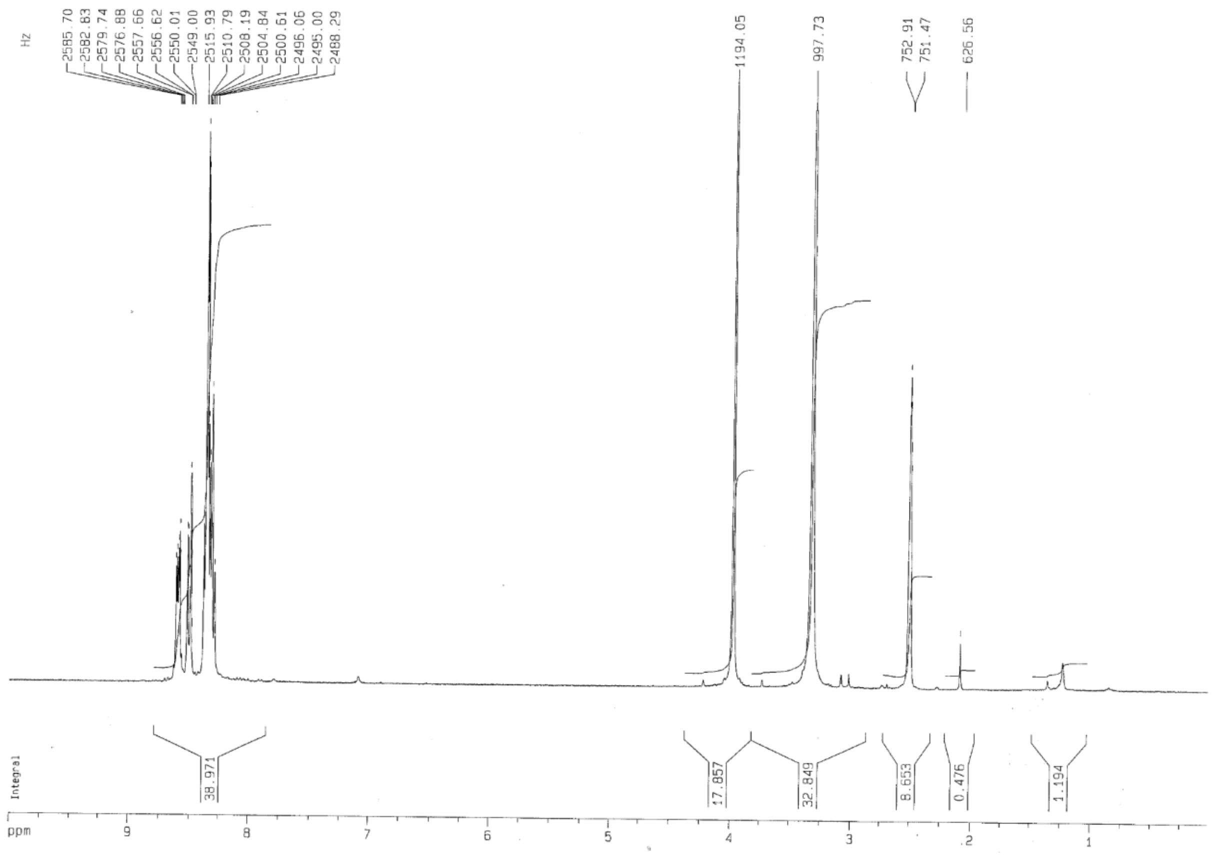

^{13}C-NMR – DMSO d6: Fragment **24**

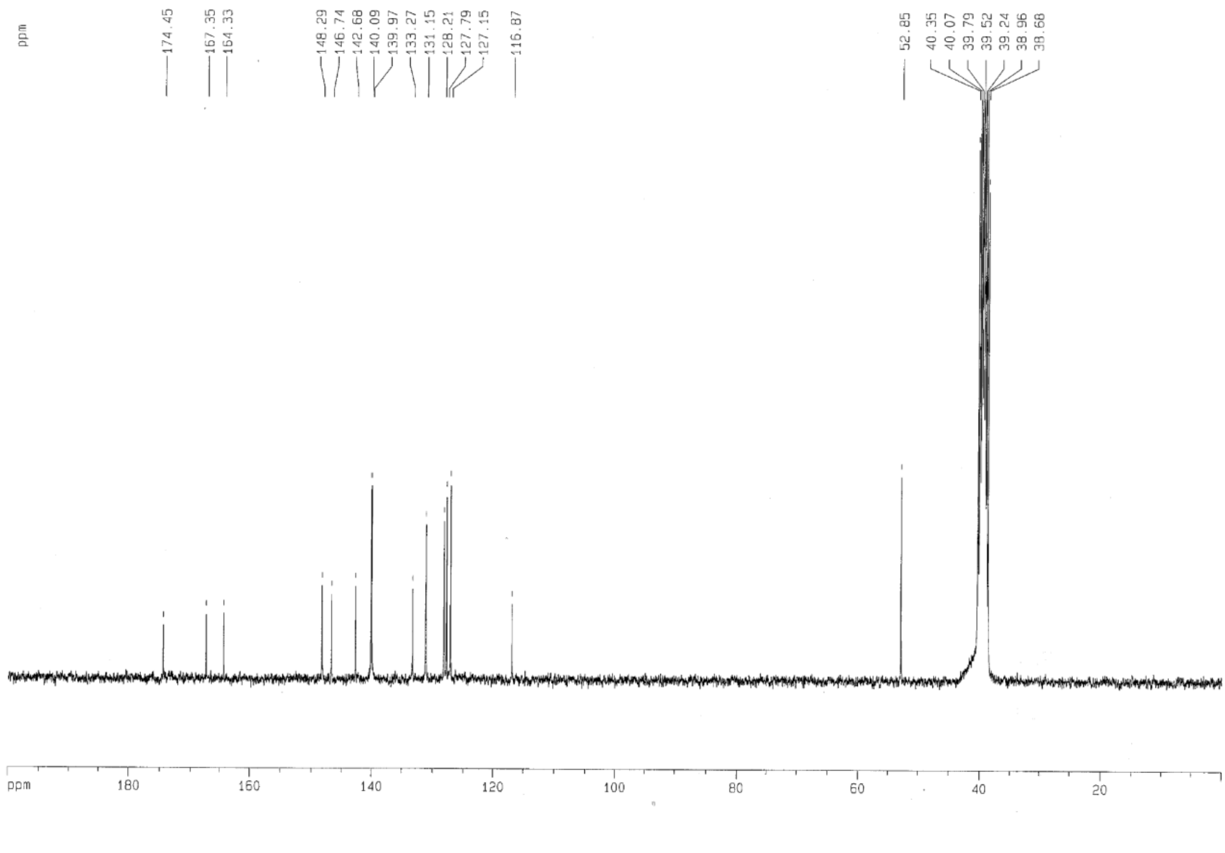

^{1}H-NMR – DMSO d6: Fragment **25**

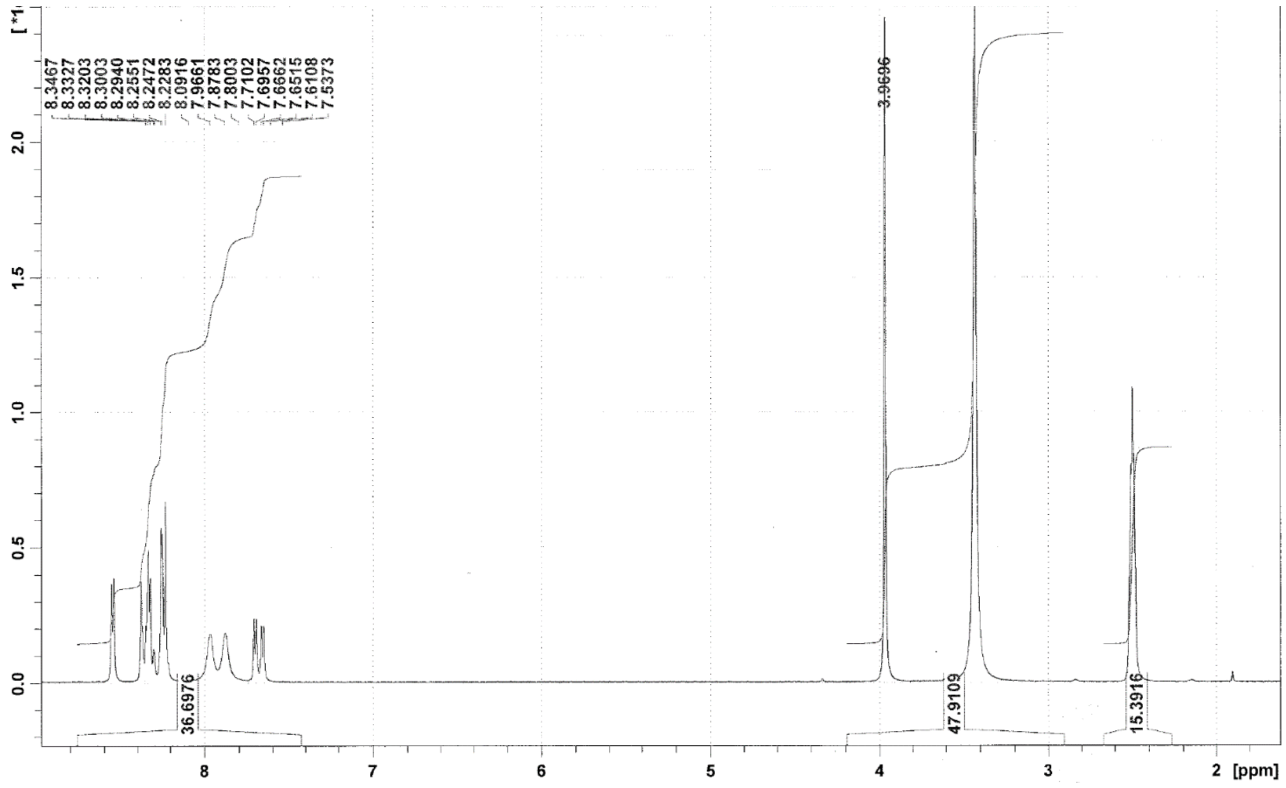

^{1}H-NMR – DMSO d6: Fragment **26**

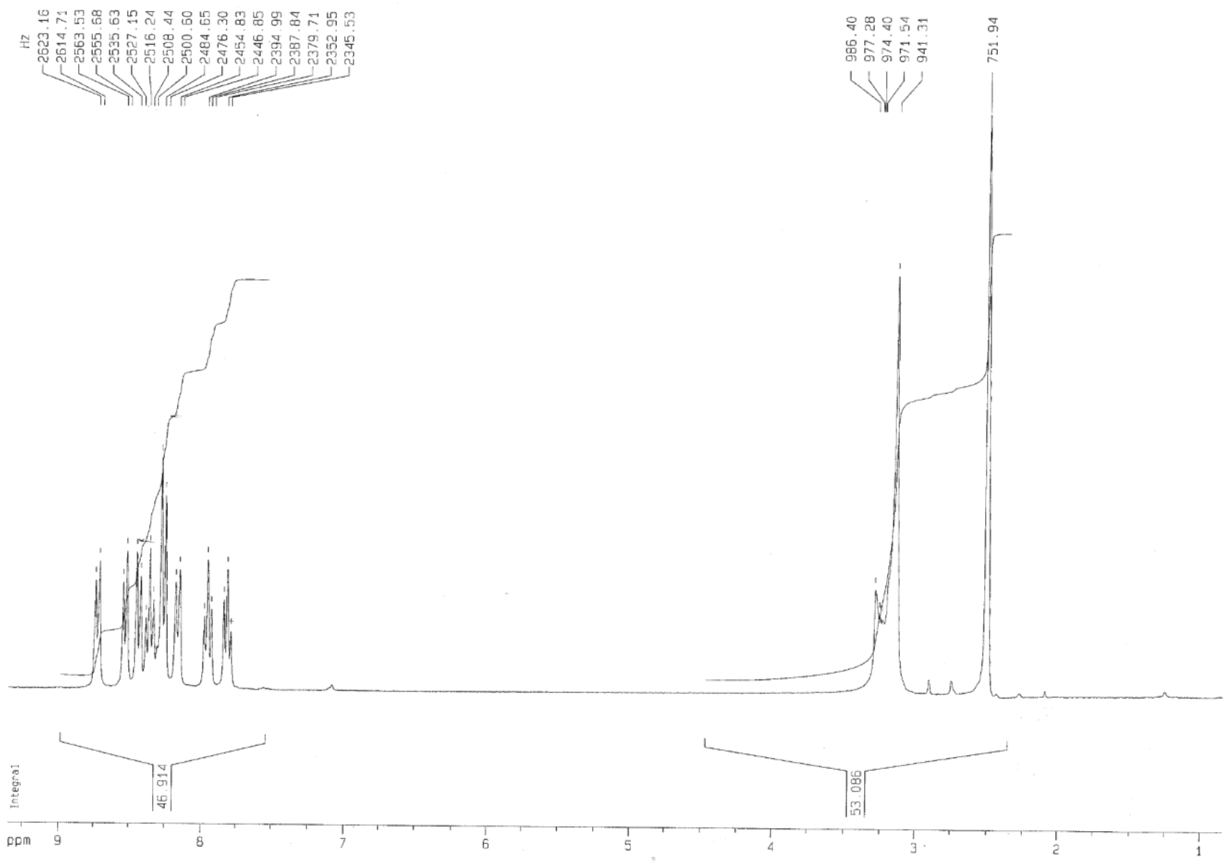

^{13}C-NMR – DMSO d6: Fragment **26**

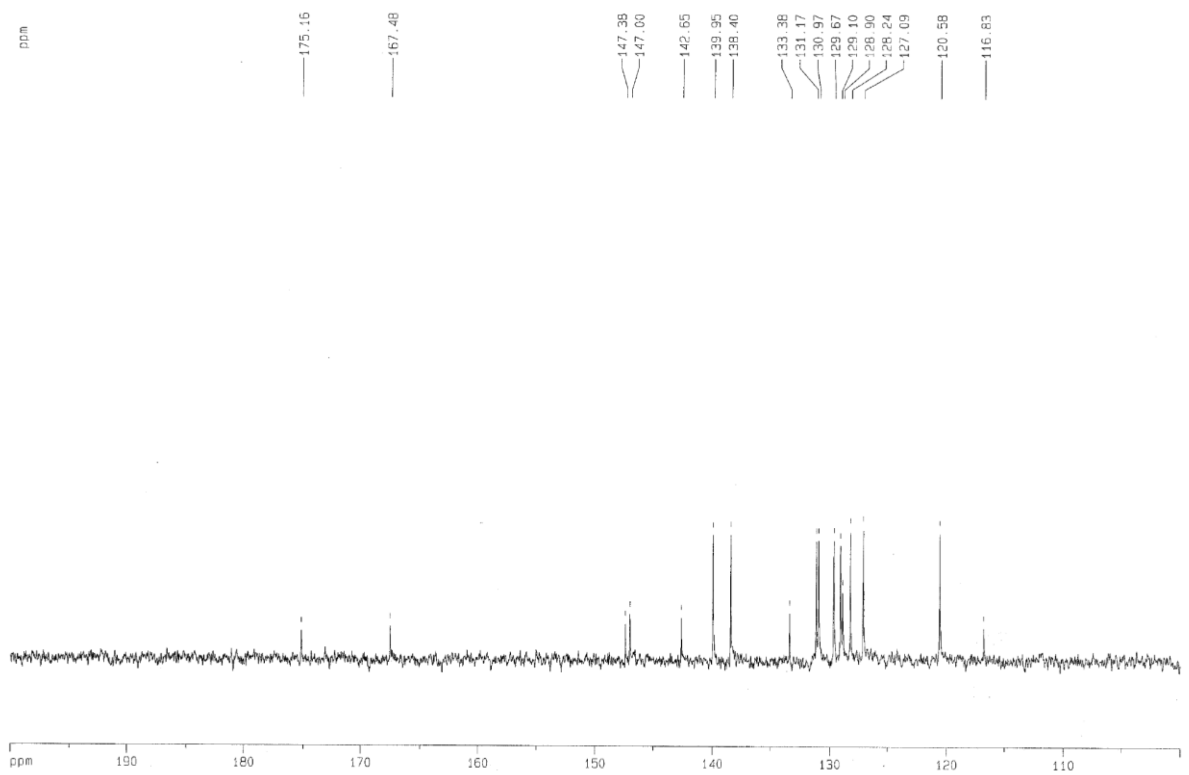

^{1}H-NMR – DMSO d6: Fragment **27**

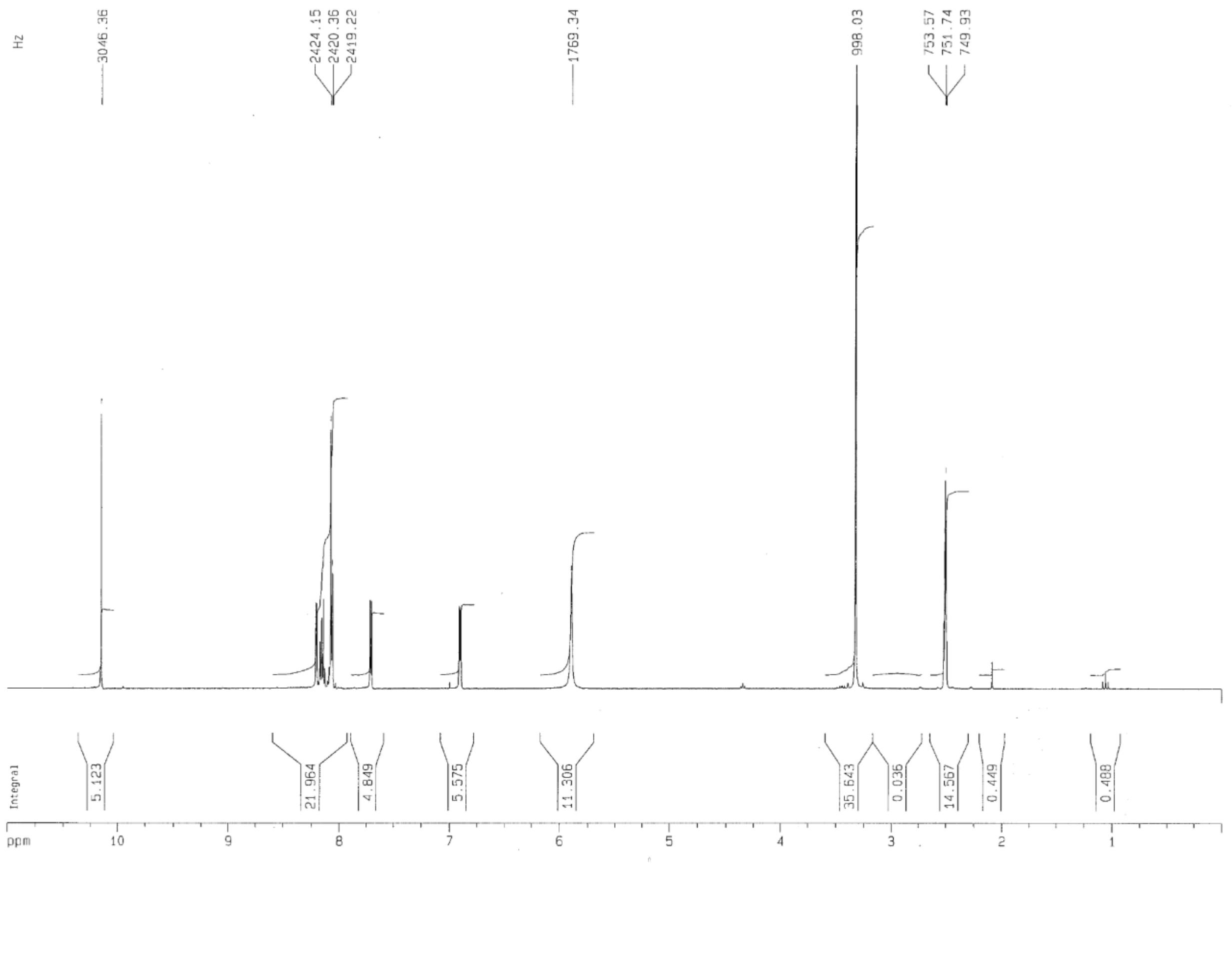

^{13}C-NMR – DMSO d6: Fragment **27**

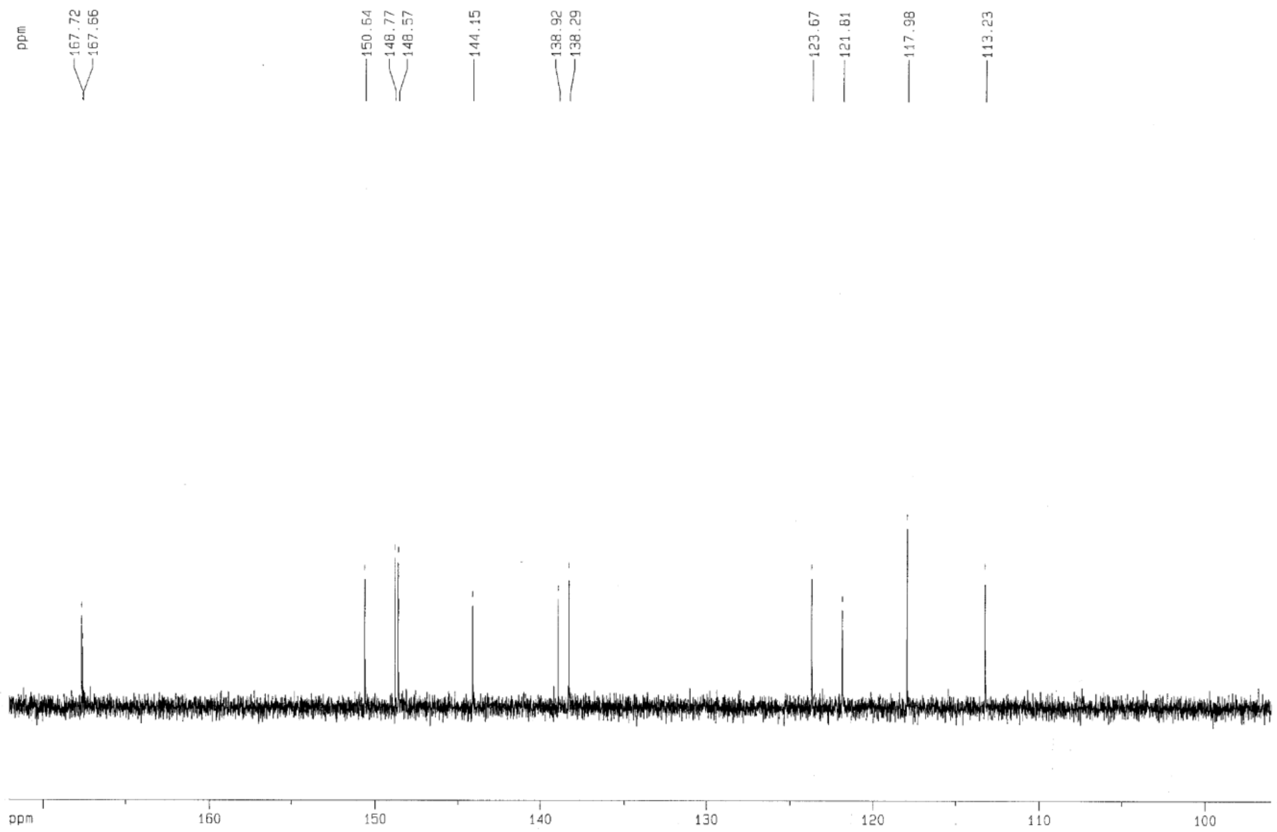

^{1}H-NMR – DMSO d6: Fragment **28**

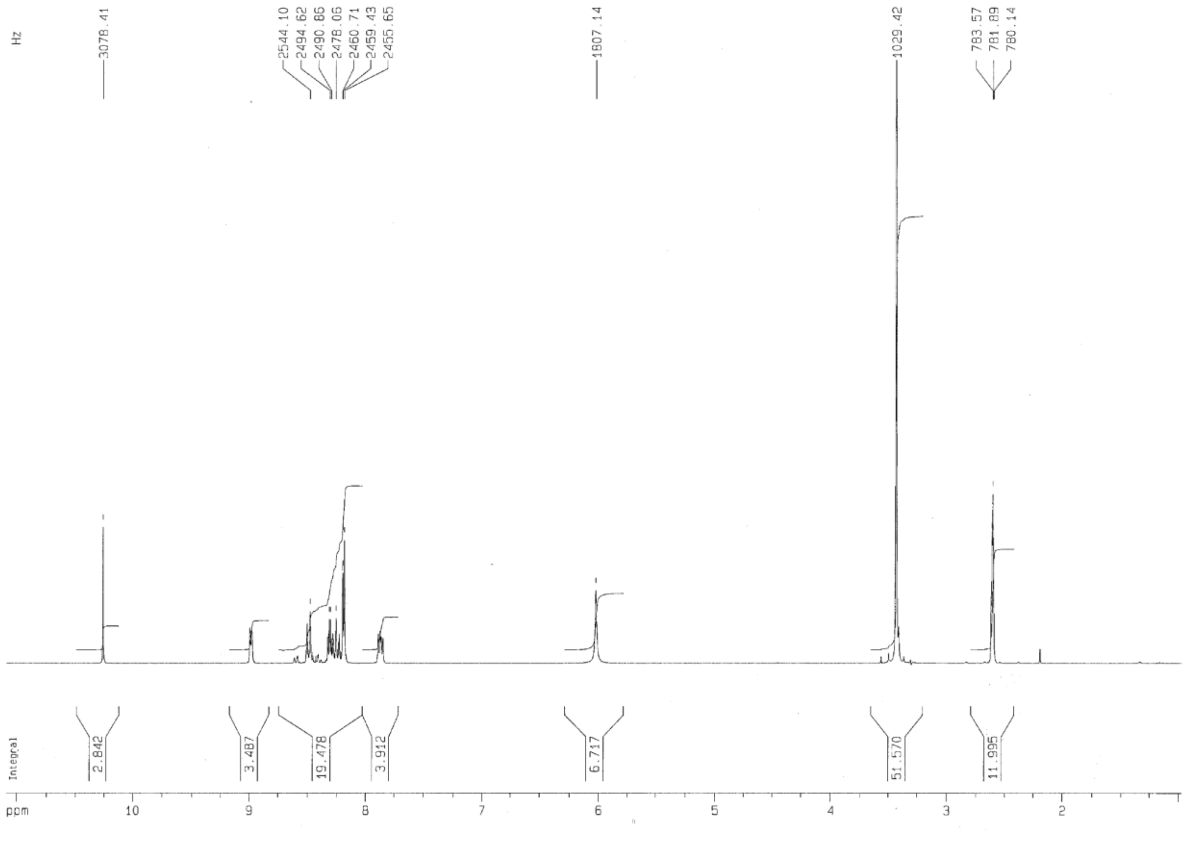

^{13}C-NMR – DMSO d6: Fragment **28**

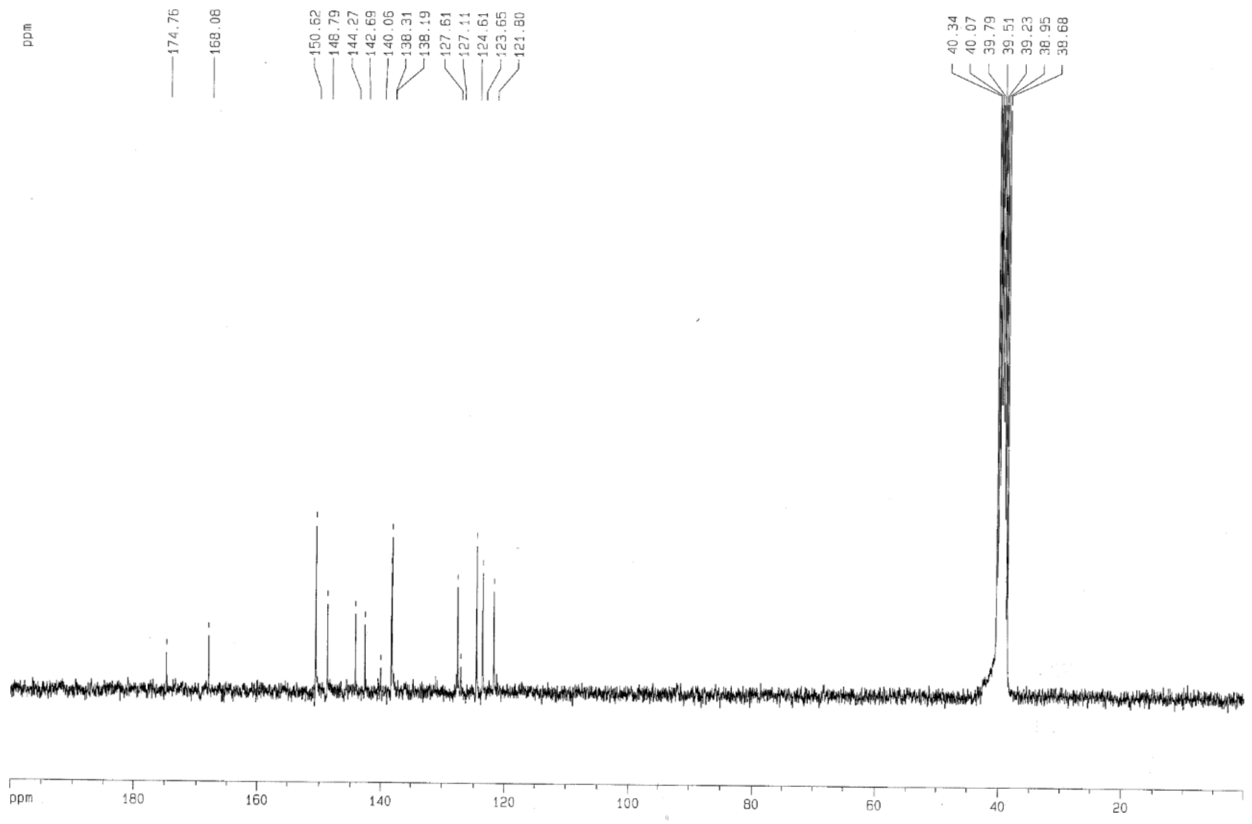

^{1}H-NMR – DMSO d6: Fragment **29**

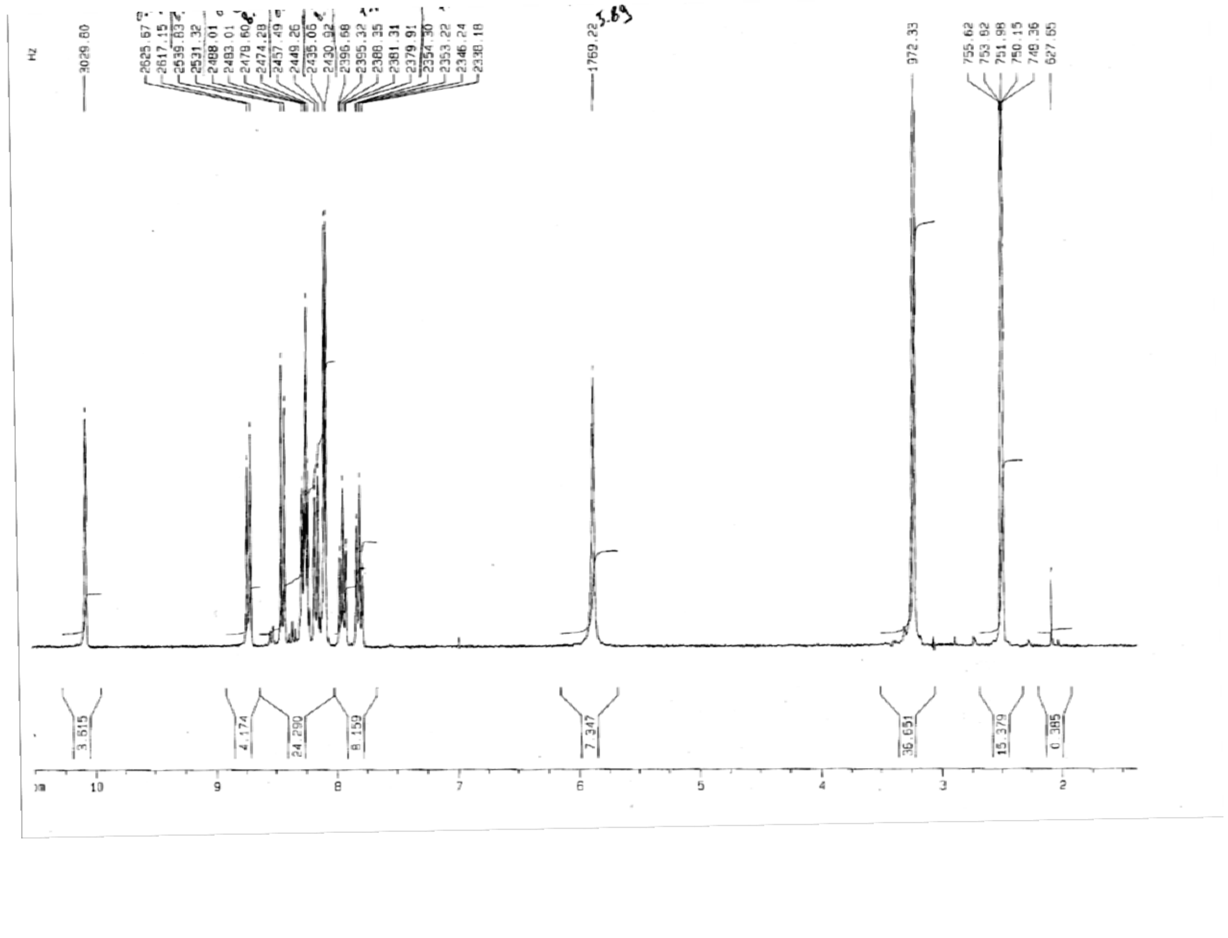

^{13}C-NMR – DMSO d6: Fragment **29**

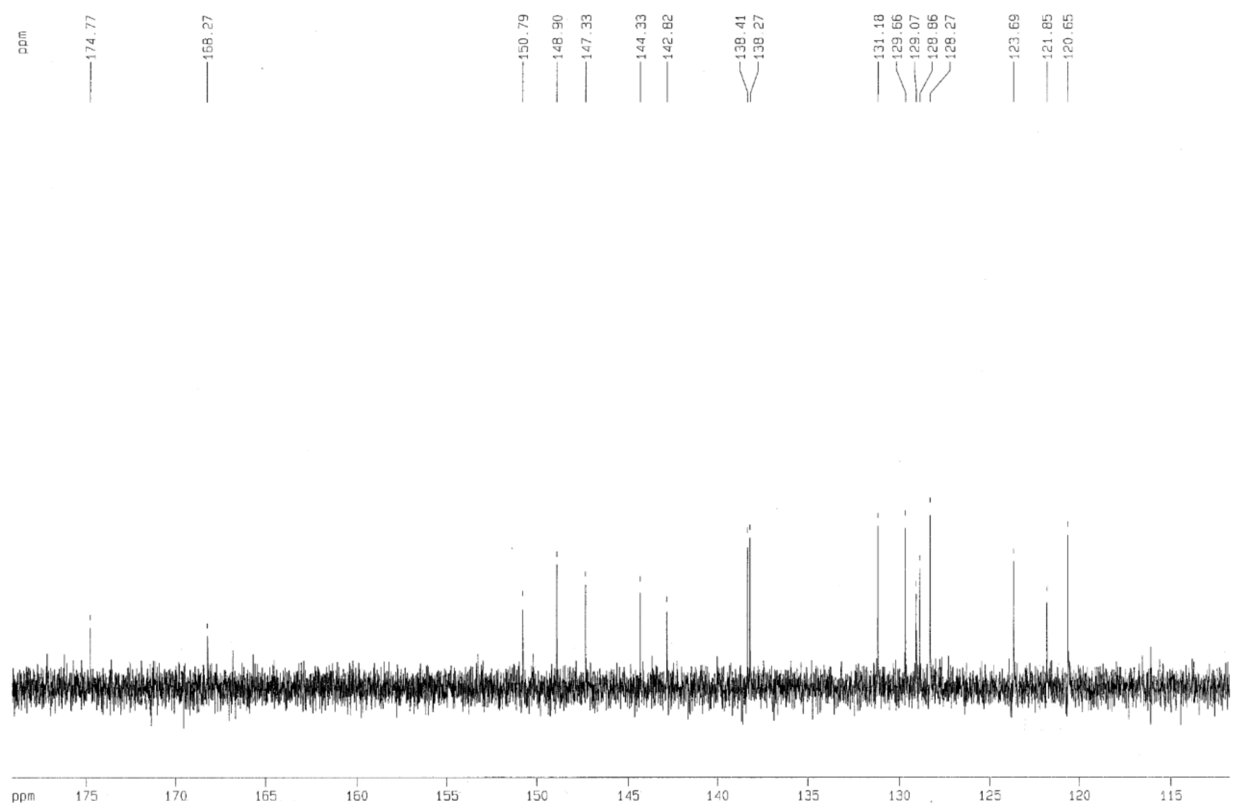

^{1}H-NMR – $CDCl_3$: Fragment **31**

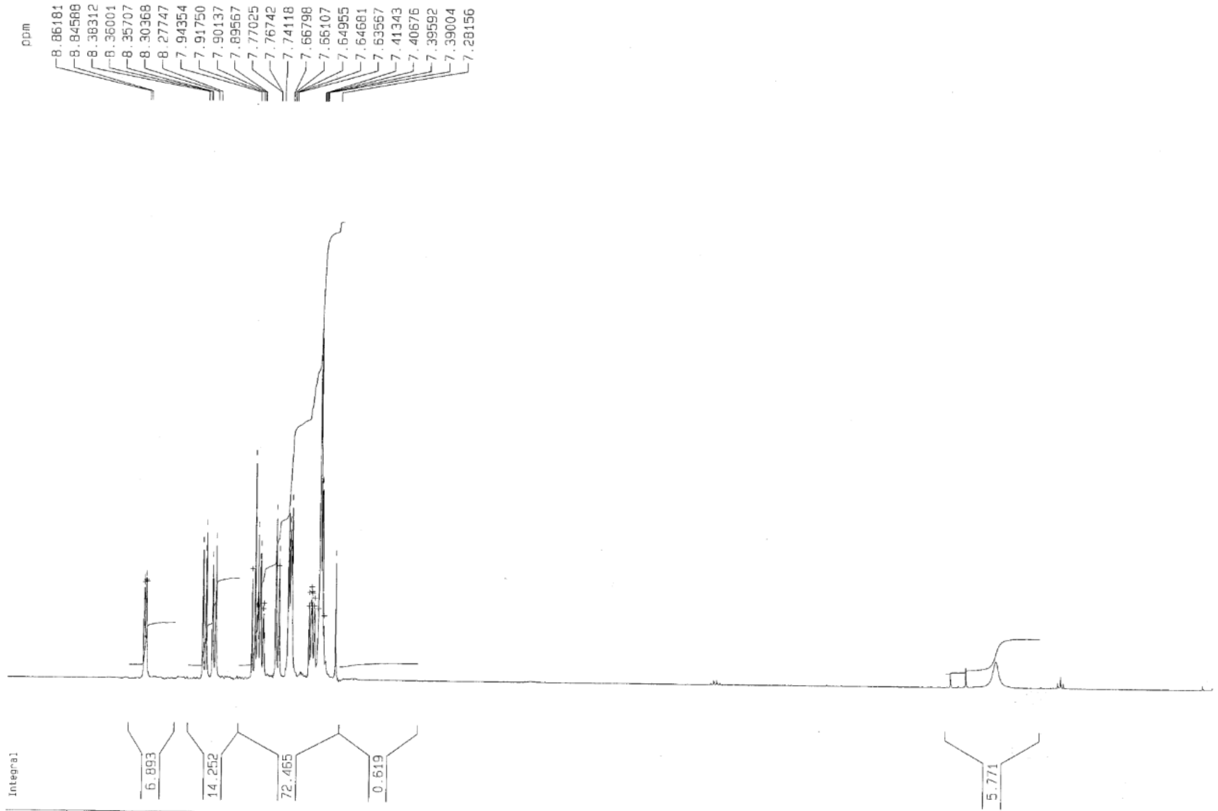

^{13}C-NMR – $CDCl_3$: Fragment **31**

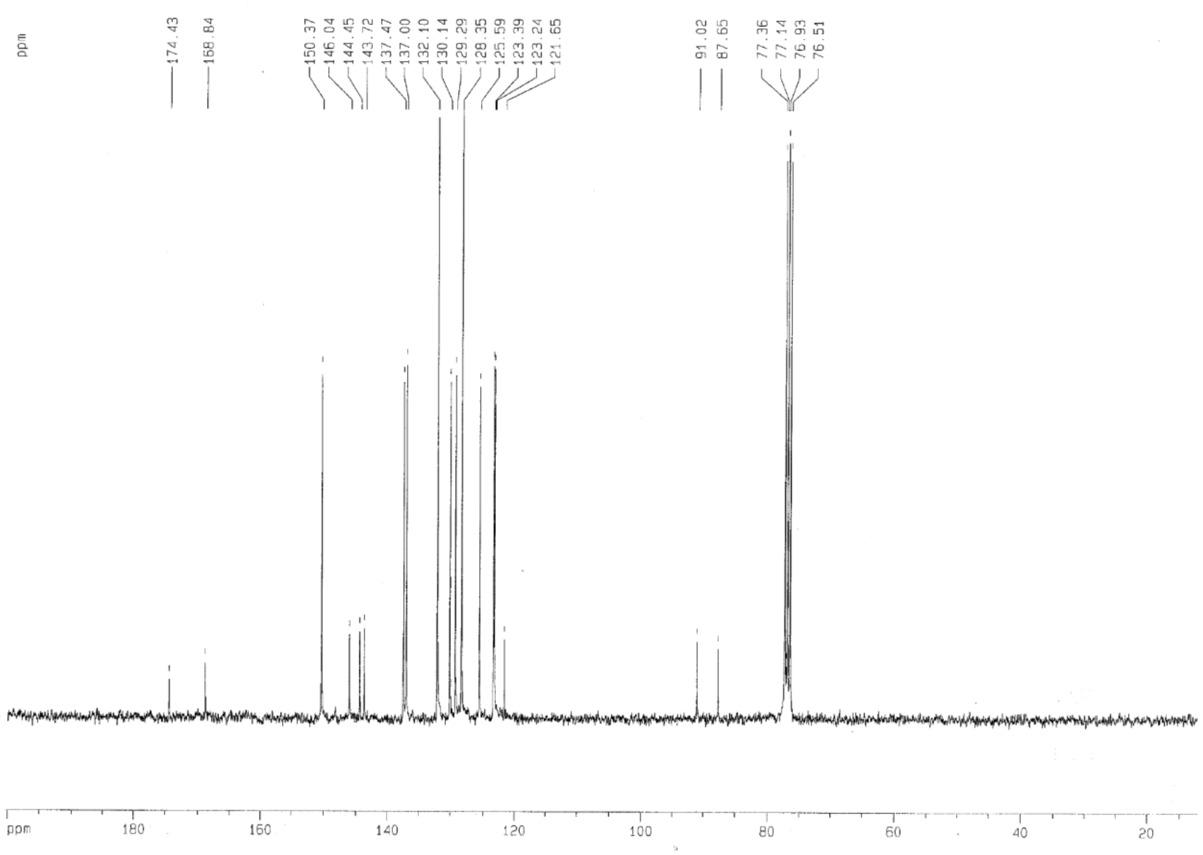

^{1}H-NMR – DMSO d6: Fragment **32**

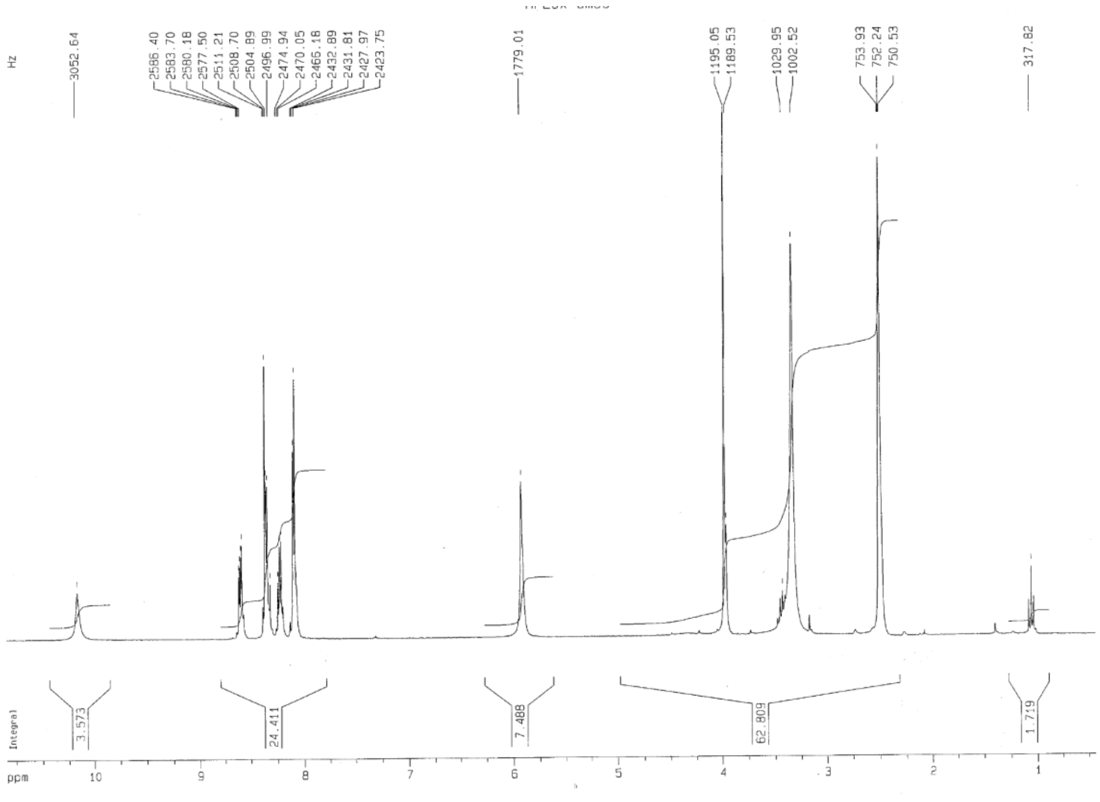

^{13}C-NMR – DMSO d6: Fragment **32**

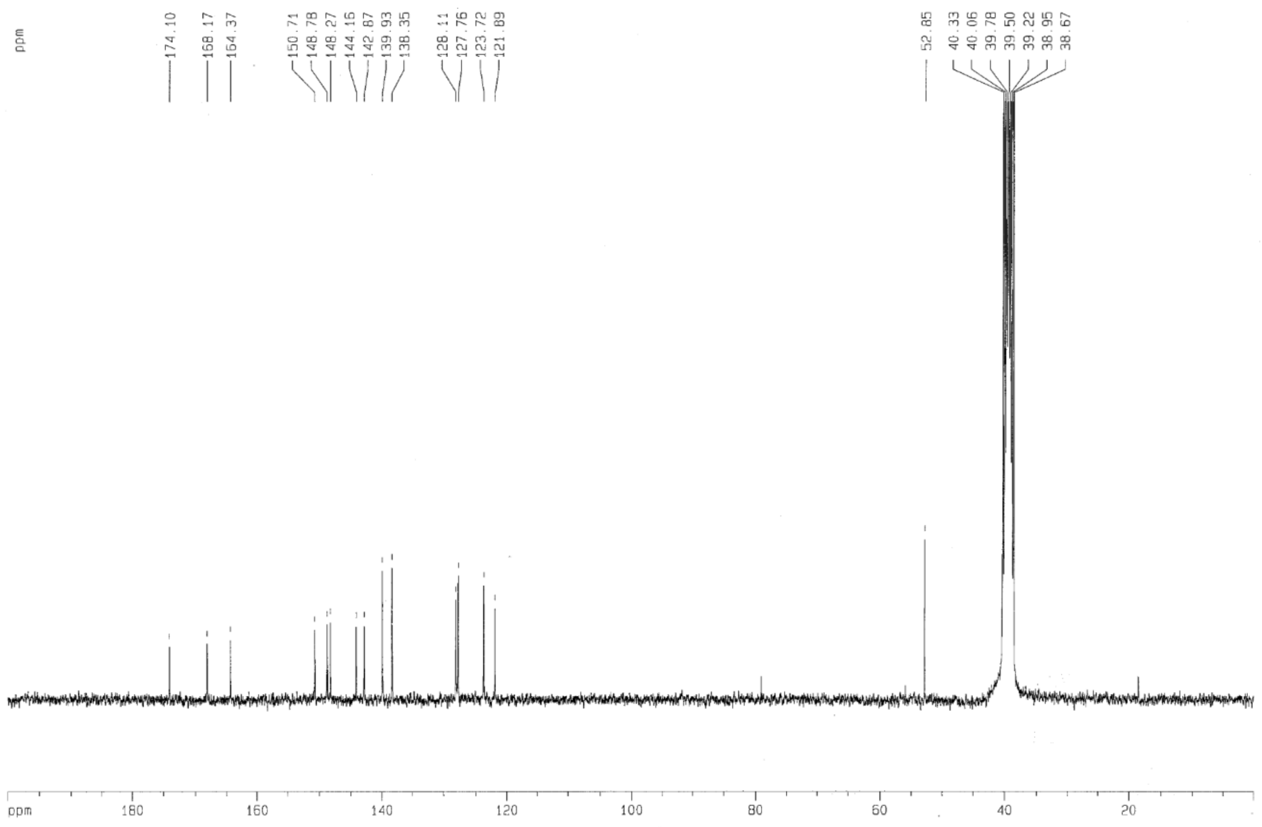

^{1}H-NMR – $CDCl_3$: Fragment **33**

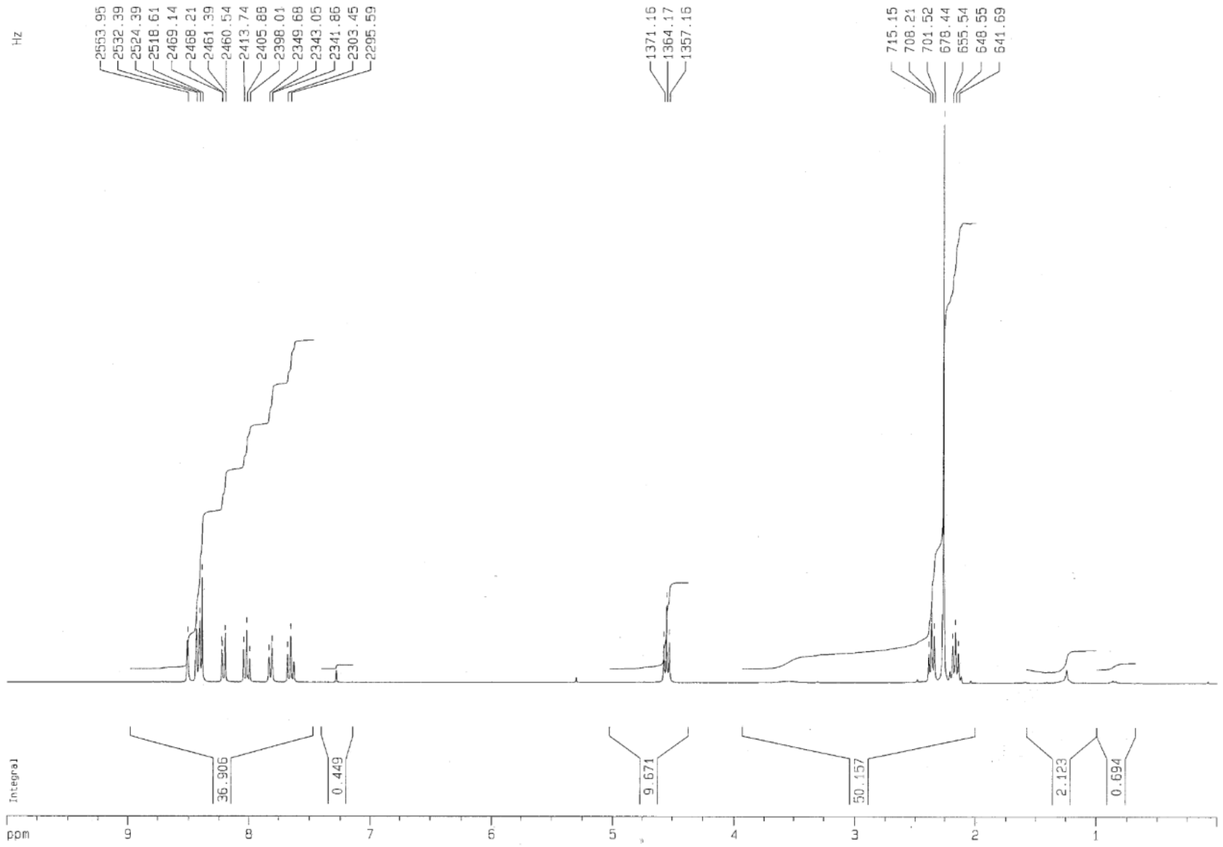

^{13}C-NMR – $CDCl_3$: Fragment **33**

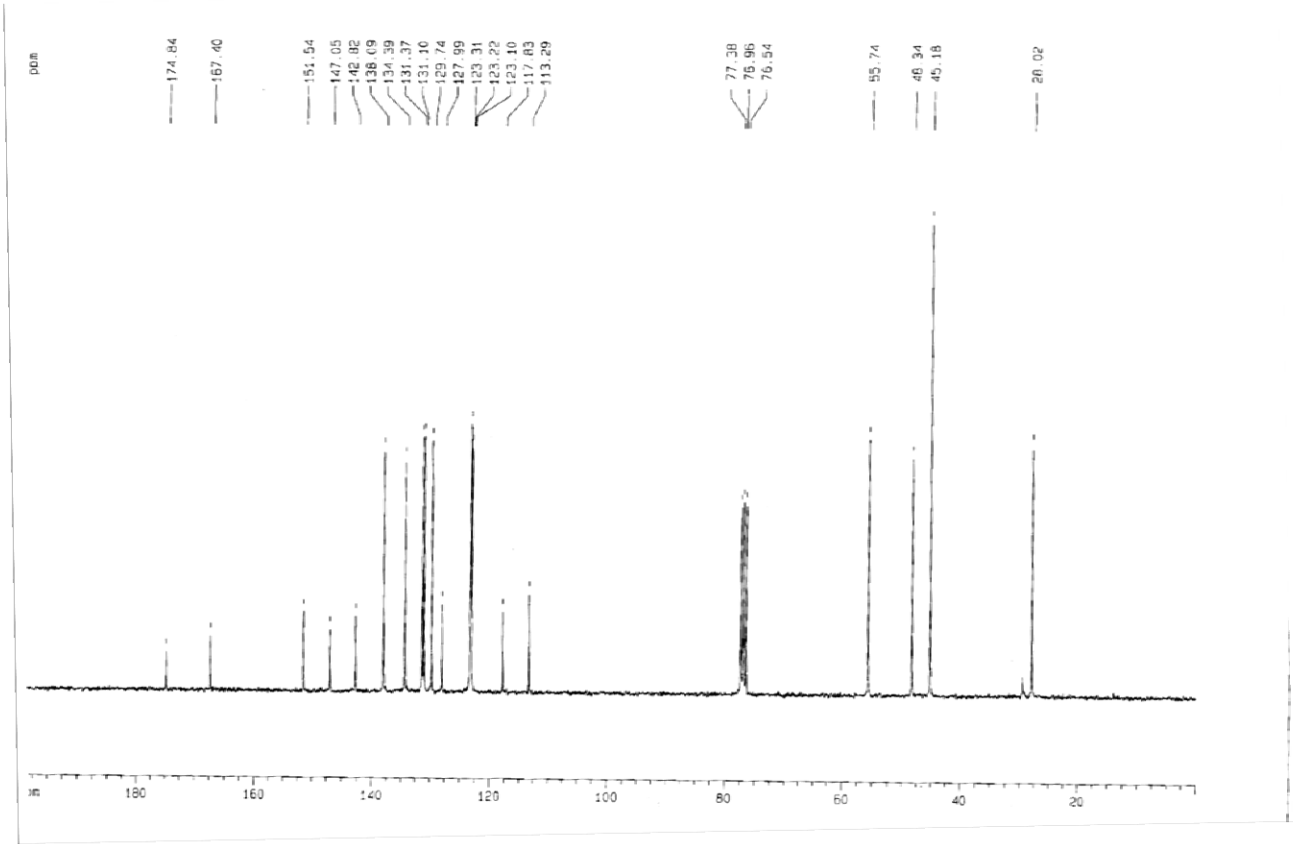

^{1}H-NMR – $CDCl_3$: Fragment **34**

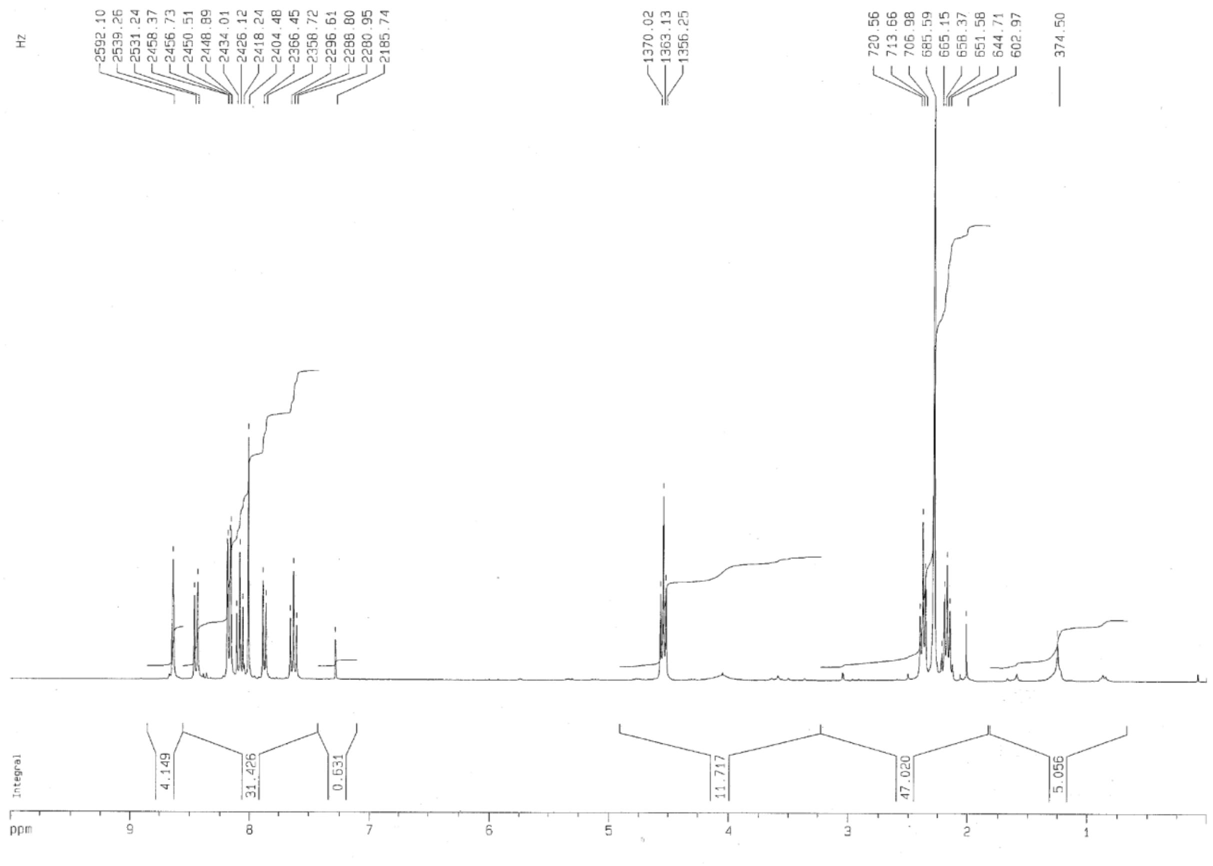

^{13}C-NMR – $CDCl_3$: Fragment **34**

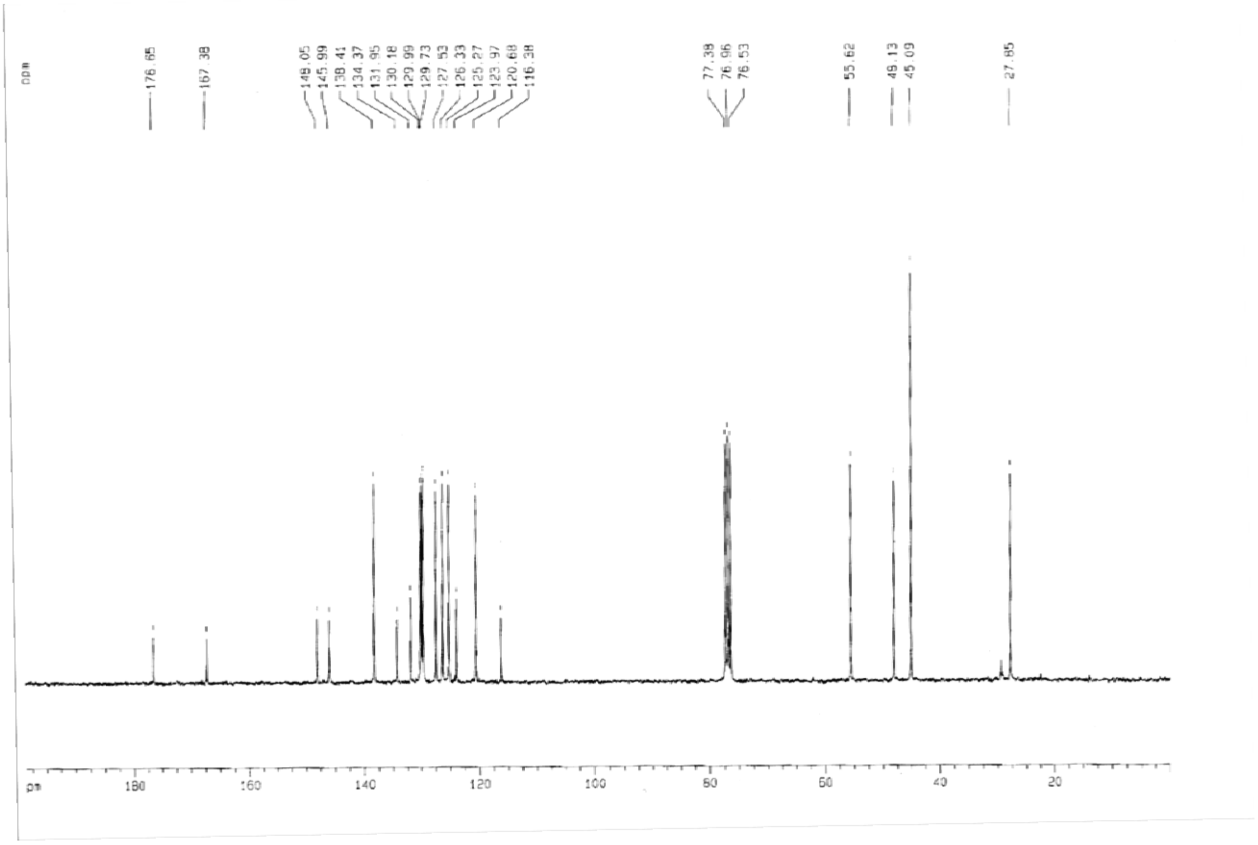

^{1}H-NMR – DMSO d6: Fragment **35**

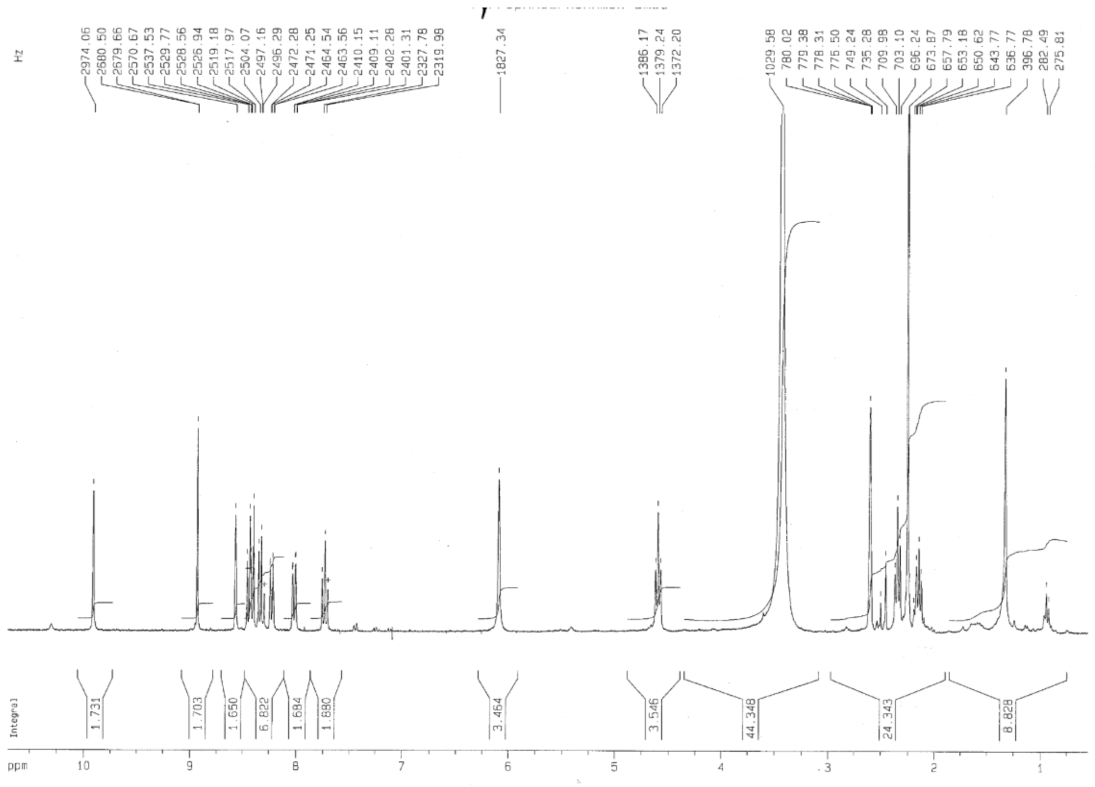

^{13}C-NMR – DMSO d6: Fragment **35**

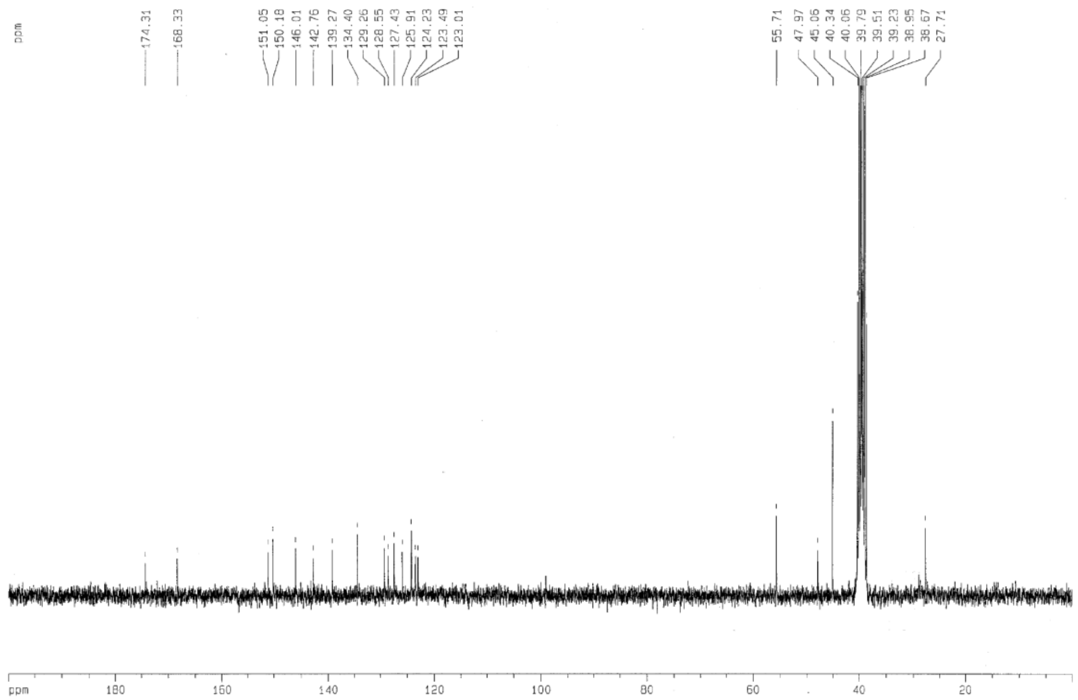

^{1}H-NMR – DMSO d6: Fragment **36**

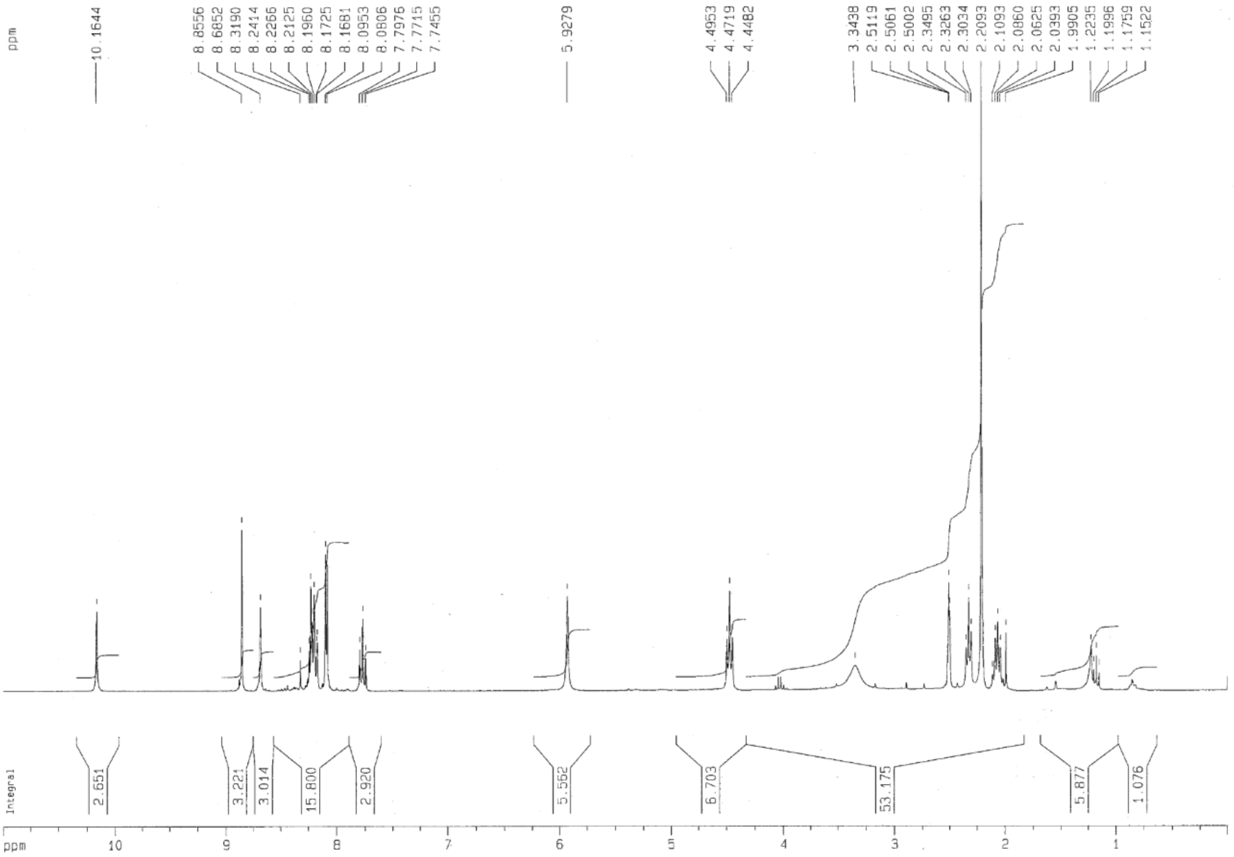

^{13}C-NMR – DMSO d6: Fragment **36**

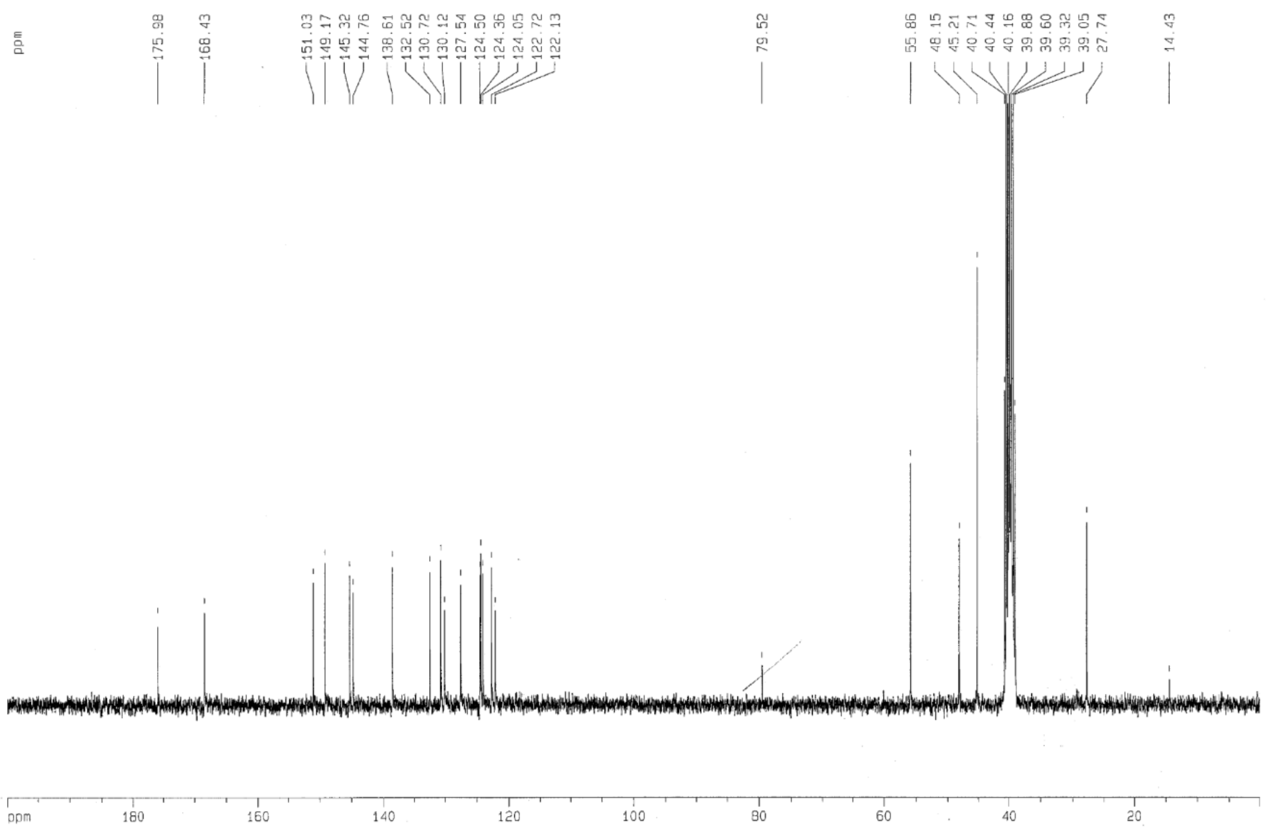

Supplement: Supplementary file 1 [file molecules-23-01874-s001.pdf]
